# Biomarkers of environmental enteric dysfunction and neurodevelopmental outcomes among children in rural Bangladesh and Kenya: a prospective cohort study

**DOI:** 10.1016/j.ajcnut.2025.05.034

**Published:** 2025-06-04

**Authors:** Gene G Ho, Beryl S Achando, Shahjahan Ali, Caitlin Hemlock, Helen O Pitchik, Christine P Stewart, Fahmida Tofail, Md Ziaur Rahman, Mohammad Alauddin, Gouthami Rao, Holly N Dentz, John P Buleti, Cecilia Nekesa, Charles D Arnold, Syeda L Famida, Md Saheen Hossen, Palash Mutsuddi, Salma Akther, Jessica A Grembi, Sophia T Tan, Sarah T Alauddin, Theodora Meerkerk, Marlene K Wolfe, Priscah Cheruiyot, Sammy M Njenga, Abul K Shoab, Mahbubur Rahman, Leanne Unicomb, Benjamin F Arnold, Patricia Kariger, Alan E Hubbard, John M Colford, Amy J Pickering, Clair Null, Stephen P Luby, Lia CH Fernald, Andrew N Mertens, Audrie Lin

**Affiliations:** 1 Division of Epidemiology and Biostatistics, School of Public Health, University of California, Berkeley, Berkeley, CA, United States; 2 Innovations for Poverty Action, Nairobi, Kenya; 3 Department of Epidemiology, Colorado School of Public Health, University of Colorado, Denver, CO, United States; 4 Institute for Global Nutrition, University of California Davis, Davis, CA, United States; 5 Nutrition Research Division, International Centre for Diarrhoeal Disease Research, Bangladesh (ICDDR,B), Dhaka, Bangladesh; 6 Department of Microbiology and Environmental Toxicology, University of California, Santa Cruz, Santa Cruz, CA, United States; 7 Department of Chemistry, Wagner College, Staten Island, NY, United States; 8 Department of Environmental Science and Engineering, Gillings School of Global Public Health, University of North Carolina at Chapel Hill, Chapel Hill, NC, United States; 9 Environmental Health and WASH, Health System and Population Studies Division, International Centre for Diarrhoeal Disease Research, Bangladesh (ICDDR,B), Dhaka, Bangladesh; 10 Department of Veterinary and Biomedical Sciences, Pennsylvania State University, University Park, PA, United States; 11 Division of Infectious Diseases and Geographic Medicine, Stanford University, Stanford, CA, United States; 12 Department of Environmental Health, School of Public Health, Emory University, Atlanta, GA, United States; 13 Kenya Medical Research Institute, Nairobi, Kenya; 14 Global Health and Migration Unit, Department of Women’s and Children’s Health, Uppsala University, Uppsala, Sweden; 15 Francis I. Proctor Foundation, University of California, San Francisco, CA, United States; 16 Division of Community Health Sciences, School of Public Health, University of California, Berkeley, Berkeley, CA, United States; 17 Department of Civil and Environmental Engineering, University of California, Berkeley, Berkeley, CA, United States; 18 Food and Agriculture Organization of the United Nations, Washington, DC, United States

**Keywords:** poor water, sanitation, hygiene, malnutrition, gut dysfunction, low- and middle-income countries (LMICs), gut–brain axis

## Abstract

**Background:**

Environmental enteric dysfunction (EED) may worsen undernutrition, with potential adverse effects on the developmental trajectories of millions of children in low-resource settings.

**Objectives:**

This study aimed to assess associations between EED biomarkers and subsequent child development.

**Methods:**

In a prospective cohort of 2646 children nested within 2 randomized trials in rural Bangladesh (*n* = 1374) and Kenya (*n* = 1272), EED was measured by markers of intestinal permeability (fecal alpha-1 antitrypsin; urinary lactulose and mannitol assessed through the dual sugar absorption test), inflammation (fecal myeloperoxidase and neopterin), and repair (fecal regenerating gene 1β). In Bangladesh, EED biomarkers were measured at ages 3 and 14 mo, whereas in Kenya, they were measured at 6 and 17 mo. Child development was measured by Communicative Development Inventories and World Health Organization (WHO) motor milestones at 1 y of age and by the Extended Ages and Stages Questionnaire at 2 y of age. We used generalized additive models to estimate associations between EED biomarkers and child development, adjusting for potential confounders and controlling for the false discovery rate (FDR).

**Results:**

In Bangladesh, higher concentrations of lactulose, mannitol, and alpha-1 antitrypsin were associated with worse subsequent child motor development outcomes. Elevated mannitol at 3 mo was associated with a lower WHO motor milestones sum score {–0.22 adjusted mean difference between the 25th and 75th percentile of mannitol distribution [(95% confidence interval (CI): −0.36, −0.08); FDR-corrected *P* value = 0.03]} and lower attainment of hands-and-knees crawling at year 1 [hazard ratio 0.74 (95% CI: 0.64, 0.86); FDR-corrected *P* value < 0.001]. In Kenya, we observed weak positive associations that were neither consistent nor significant after FDR correction.

**Conclusions:**

Higher concentrations of biomarkers of intestinal permeability were associated with poor child motor development in Bangladesh. These relationships were not replicated in the Kenyan cohort. The associations between EED and child neurodevelopment vary across geographic contexts, highlighting the need for further research to determine the generalizability of these findings.

## Introduction

A child’s developmental trajectory can have lasting impacts throughout adulthood[[1], [2], [3]]. Ensuring that children are able to attain their academic and economic potential is vital to ending the intergenerational cycle of poverty, especially in low- and middle-income countries (LMICs) [3,4]. For the 250 million children aged <5 y who are at risk of not reaching their full developmental potential, the contributors to poor neurodevelopment have not been fully elucidated [2,5]. Poor neurodevelopment is thought to be caused in part by malnutrition, lack of a stimulating environment, and infection and is marked by deficits in social–emotional and cognitive functions [2,5]. A child’s first 1000 d (from conception to age 2 y) is a critical period when the brain is more sensitive to environmental conditions and can have long-lasting implications for their social and behavioral development [[6], [7], [8], [9]].

One way environmental exposures may affect child development is through environmental enteric dysfunction (EED), a widespread syndrome with no formal clinical definition observed in low-resource settings [[10], [11], [12], [13]]. It has been hypothesized that inadequate access to water, sanitation, and hygiene (WASH) could lead to recurrent exposure to fecal pathogens and enteric infections resulting in lymphocytic infiltration, reduced villus height, and increased crypt depth [13]. This restructuring of the small intestinal architecture leads to reduced absorptive capacity and impaired barrier function, resulting in increased microbial translocation and systemic inflammation [13,14].

Ideally, exposure to infection would be minimized through access to WASH infrastructure [15]. In areas where this infrastructure is lacking, to mount an effective defense against repeated infections, the nutritional requirements of the immune system may be higher than normal [16,17]; this pathophysiology results in a vicious cycle, where the nutritional needs of the immune system remain unmet due to the impaired absorptive capacity of the small intestine, further exacerbating EED and undernutrition [17]. Thus, several studies have reported a link between EED biomarkers, undernutrition, and poor linear growth [10,[18], [19], [20]]. In addition to increasing undernutrition, EED may disrupt the gut–brain axis, the bidirectional signaling pathways between the gut and the brain, which could lead to impaired child neurodevelopment in LMICs [16,21].

Although a biopsy is the gold standard to measure EED, it is an invasive and expensive procedure, and not applicable for population-based studies [10,11]. Thus, biomarkers of intestinal permeability [e.g., fecal alpha-1-antitrypsin and urinary lactulose and mannitol (LM)], inflammation (e.g., fecal myeloperoxidase and neopterin), and repair [e.g., fecal regenerating gene 1β (REG1B)] are typically used as surrogate measurements of EED [10,18,[22], [23], [24]]. Previous EED studies have assessed the L:M ratio, with a higher ratio being indicative of gut dysfunction. However, studies suggest that LM are correlated, indicating that LM likely traverse the gut barrier through the same paracellular pathways [24,25]. This emerging evidence has called into question the clinical interpretation of the L:M ratio and has suggested that future studies should report LM measurements separately [24].

Few studies have investigated EED, which encompasses both gut dysfunction and systemic inflammation, and its potential association with neurodevelopment, and the results are mixed [[26], [27], [28], [29], [30]]. Notably, studies in Bangladesh found that higher levels of systemic inflammation, as measured by acute phase proteins and proinflammatory immune markers, were associated with lower neurodevelopmental scores [27,29]. Similarly, a study in Zambia found that higher markers for systemic inflammation and gut barrier dysfunction were associated with poor developmental outcomes [31]. However, a study in Tanzania reported an association between elevated EED biomarkers and better neurodevelopment, potentially through an immune pathway with components previously implicated in neuroplasticity and neurogenesis [28]. A study among HIV-exposed uninfected Tanzanian infants found that acute phase proteins were negatively associated with neurodevelopment, whereas markers of enterocyte damage were positively associated with neurodevelopment [30]. Given the inconclusive evidence, the mechanisms through which EED pathophysiology influences neurodevelopment remain poorly understood.

The WASH Benefits study was a pair of cluster-randomized controlled trials examining nutrition, drinking water, sanitation, and handwashing (WSH) interventions in rural Bangladesh and Kenya [32,33]. The Bangladesh trial reported reductions in enteric viral and parasitic infections [[34], [35], [36]], early reductions in intestinal permeability and inflammation [37], and improvements in linear growth [32] and child neurodevelopment [38] in the intervention arms. In Kenya, the interventions reduced *Ascaris* infections [39], improved linear growth [33], and improved child motor function in the first year but not the second year [40]. Building on these findings, this prospective cohort study aimed to assess the associations between markers of EED (neopterin, myeloperoxidase, alpha-1 antitrypsin, lactulose, mannitol, and REG1B) and subsequent child neurodevelopment among WASH Benefits participants in Kenya and Bangladesh. We hypothesized that higher levels of EED biomarker concentrations would be associated with lower child development scores.

## Methods

### Study design

The WASH Benefits study was a pair of cluster-randomized controlled trials conducted in rural villages in the Gazipur, Mymensingh, Tangail, and Kishoreganj districts of Bangladesh (NCT01590095) and in Bungoma, Kakamega, and Vihiga counties in Western Kenya (NCT01704105) [32,33]. The studies sought to evaluate the effect of improved WSH, and nutrition on child growth and diarrhea, the primary outcomes of the trial. In the Kenya trial, study enrollment occurred between November 2012 and May 2014. In the Bangladesh trial, study enrollment occurred between May 2012 and July 2013. Participants were randomly assigned to either the double-sized control arm or 1 of the 6 intervention arms from the parent study: water, sanitation, handwashing, a combined WSH arm, nutrition, or a combined nutrition and WSH (N+WSH) arm. Interventions were implemented for ∼2 y.

### Interventions at the Bangladesh study site

The interventions consisted of: water treatment and safe storage vessel; sanitation (child potties, sani-scoop hoes to remove feces, and a double pit latrine with a hygienic water seal); handwashing (handwashing stations near the latrine and kitchen, including soapy water bottles and detergent soap); and nutrition (lipid-based nutrient supplements). The nutrient supplements contained ≥100% of the recommended daily allowance of 12 vitamins and 9 minerals with 9.6 g of fat and 2.6 g of protein daily for children aged 6–24 mo. The nutrition intervention included recommendations on maternal nutrition and age-appropriate recommendations on infant feeding practices. For the first 6 mo, community health promoters visited households in the intervention arm at least once a week, then the schedule changed to biweekly visits thereafter. Households in the control arm did not receive any interventions or promoter visits.

### Interventions at the Kenya study site

The interventions consisted of: chlorinated drinking water treatment (chlorine dispensers at water sources and point-of-use water treatment); sanitation (improved latrines, child potties, and sani-scoops); handwashing (handwashing stations including soapy water); and nutrition (lipid-based nutrient supplements and dietary recommendations). The nutrition intervention in Kenya was the same as the intervention in Bangladesh. The active control arm received monthly visits from community health promoters, for ∼10 min each time, and the intervention groups received visits that lasted 45–60 min.

### EED substudy

**FIGURE 1:**
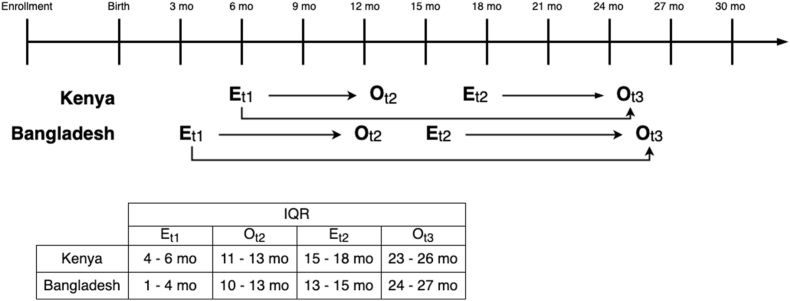
Study timeline of environmental enteric dysfunction (EED) exposure and child development outcome measurements. E, exposure (EED biomarkers); O, outcome (child development). To simplify analyses, the exposures measured at follow-up 3 were considered “concurrent” and were not assessed.

The original EED substudy examined the effects of the interventions on EED in a subsample of children, who underwent intensive biological sampling [37]. This study only included the participants who were enrolled in the previous EED substudy. The EED substudy only included children in the control (active control arm in Kenya and there was only 1 passive control arm in Bangladesh); nutrition; combined WSH; and combined nutrition, water, sanitation, and handwashing arms (N+WSH). Children enrolled in the EED substudy participated in 3 visits for data and biological specimen collection, beginning shortly after intervention initiation and continuing through March 2016 in Bangladesh and April 2016 in Kenya (Figure 1). For this substudy, we measured exposures (EED biomarkers) at the first 2 time points (E_t1_ and E_t2_); the third exposure time point was a concurrent measurement and, therefore, not used in the current study. The median age of EED measurement was 6 mo for follow-up 1 (E_t1_) and 17 mo for follow-up 2 (E_t2_) in Kenya. The median age of EED measurement was 3 mo for follow-up 1 (E_t1_) and 14 mo for follow-up 2 (E_t2_) in Bangladesh. The child neurodevelopment outcomes were measured at 2 visits in all study children [38,40]. In Kenya, the median age of child neurodevelopment measurement was 12 mo for year 1 (follow-up 2; O_t2_) and 25 mo for year 2 (follow-up 3; O_t3_). In Bangladesh, the median age of child neurodevelopment measurement was 11 mo for year 1 (follow-up 2; O_t2_) and 26 mo for year 2 (follow-up 3; O_t3_).

### Sample size

The total sample size for this EED and neurodevelopment substudy consisted of 2646 children (1374 in Bangladesh and 1272 in Kenya). This substudy used the same subsample as the original EED study. Sample size and power calculations for the original EED substudy were previously published [37]. Power calculations from the original EED substudy included a sample size of 1500 children at each study site. With a 5% 2-sided type 1 error, 54 clusters enrolled per arm, and 7 children measured per cluster, along with a cluster-level intraclass correlation coefficient of 0.15, the original EED substudy achieved 80% power to detect a difference of 0.26 SDs in standardized log EED biomarkers between any 2 arms.

### Participants

In Bangladesh, pregnant females in their first 2 trimesters were enrolled in this study along with their in-utero children. They were eligible for inclusion if the females planned to live in the same study village for the subsequent 2 y. Similarly, in Kenya, pregnant females were eligible for inclusion if they planned to live in the same residence for the following 2 y. Expectant females were eligible for enrollment if they were in their second or third trimester of pregnancy. Additional details regarding the enrollment criteria have been published previously [32,33]. The primary caregivers of all children provided informed consent. The study procedures for the Bangladesh study were reviewed and approved by the human subjects committees at the International Centre for Diarrhoeal Disease Research, Bangladesh (ICDDR,B), the University of California, Berkeley, and Stanford University. The Kenya study protocol was approved by the human subjects committees at the Kenya Medical Research Institute (KEMRI), the University of California, Berkeley, and Stanford University.

### Bangladesh and Kenya study procedures

#### Exposures: EED biomarker measurements

Fecal samples from participants in Bangladesh and Kenya were collected without fixative, transported on dry ice, and stored at –80ºC at ICDDR,B and KEMRI. The fecal samples from the Kenya cohort were shipped on dry ice to ICDDR,B. Fecal samples from both countries were analyzed at the same ICDDR,B lab. The ICDDR,B lab utilized ELISA kit protocols to measure fecal alpha-1 antitrypsin (BioVendor; detection limit: 1.5 ng/mL), myeloperoxidase (Alpco; detection limit: 1.6 ng/mL), neopterin (GenWay Biotech; detection limit: 0.7 nmol/L), and regenerating gene 1β (REG1B; TechLab) [37]. Samples were analyzed in singlicate. The initial dilutions were as follows: 1:500 for myeloperoxidase, 1:75 in 0.9% saline for neopterin, 1:25,000 for alpha-1-antitrypsin, and 1:40,000 for REG1B. The standard curve and high and low kit controls were run in duplicate on each plate. Samples were considered out-of-range if their values fell outside the standard curve and were subsequently rerun at higher or lower dilutions. The intra-assay coefficient of variation (CV) for the assays ranged from 5.7% to 8.8%. Alpha-1 antitrypsin is a measure of intestinal permeability, whereas myeloperoxidase and neopterin are indicators of intestinal inflammation [10]. Finally, REG1B is a measure of intestinal repair [18].

In addition to alpha-1 antitrypsin, intestinal permeability was assessed using the dual sugar absorption test, which measures the differential urinary excretion of LM [24]. Each child was administered a solution of lactulose (250 mg/mL; USP, Coghlan Group) and mannitol (50 mg/mL; USP, VWR) (LM) solution, at 2 mL/kg with a maximum of 20 mL. A single-void, pre-LM and continuously pooled 5-h post-LM urine specimens were collected and analyzed for LM recovery using HPLC with electrospray ionization tandem mass spectrometry (HPLC-ESI MS/MS) at Wagner College, Staten Island, New York following previously published protocols [37].

Briefly, all standards (^13^C-labeled mannitol and ^13^C-labeled lactulose), lactulose, mannitol, acetonitrile (ACN), ammonium acetate, and water were LC-MS grade (Sigma-Aldrich). Separate stock solutions of lactulose, mannitol, and internal standards were prepared by dissolving standards in an ACN/water (90:10) mixture. The concentration of each stock standard solution of lactulose and mannitol was adjusted to 10 μg/mL. Tandem mass spectrometry calibration solutions for each analyte were prepared by serial dilution of the stock standard with ACN/H_2_O. The concentration of internal standard in each calibration standard, sample, and quality control (QC) samples was maintained at 0.50 μg/mL for mannitol and 2.00 μg/mL for lactulose. Blank urine samples (collected in the absence of mannitol and lactulose dosage) were spiked with serially diluted standards for method validation and QC checks on sample runs.

Each 50 μL-aliquot of urine sample was diluted with 500 μL of LC-MS grade water (10-fold dilution) and centrifuged at ambient temperature for 3 min at 5000 × *g*. Then, 25 μL of prepared urine sample was diluted with 100 μL of stock internal standard and 50 μL of ACN. The calibration standards and QC samples were prepared in the same manner. All solutions were mixed for 10 min using a rotary platform.

The HPLC-ESI MS/MS system was composed of the Acquity UPLC with degasser, autosampler, column oven (Waters Corporation). The ESI MS/MS (API 3200 Model, AB Sciex, DC) with turbo ion probe electrospray ionization was operated at 600°C in negative ion mode. The chromatographic column used was ZIC-HILIC (Nest Group), 250 mm ×2.1 mm, 5 μm with pore size 200 Å with a flow rate of 0.400 mL/min at 30°C (column temperature). The separation of mannitol and lactulose was achieved through gradient elution involving mobile phase B (5 mM NH_4_OAc in ACN/H_2_O, 90/10, v/v) which was kept at 90% from 0 to 1 min, then changed to 85% at 10 min, followed by a 3-min washing step with 100% of mobile phase A, and 2 equilibrations with 90% B. Mobile phase A was 5 mM NH_4_OAc in H_2_O/ACN, 90/10, v/v. A sample injection volume of 15 μL was employed.

To determine the limits of detection for the LM assay, calibration curves for each analyte were constructed by plotting the ratio of analyte peak area to internal standard peak area against analyte concentration. Each calibration solution was injected and analyzed 3 times. Calculations of limits of detection and limits of quantitation were determined from the SD of the constant term of calibration equation and slope of calibration curve. Repeatability and precision of the method were tested with prepared spiked samples at 3 levels of concentration. The method detection limit (MDL) values for each analyte were calculated by multiplying the mean of spiked sample SDs by Student’s *t*-value. Method of quantitation limit values were obtained by multiplying MDL by 3. MDL estimated from replicate analysis of standards was 0.314 μg/mL for mannitol and 0.200 μg/mL for lactulose. Method quantitation limit for mannitol and lactulose were estimated as 0.942 μg/mL and 0.600 μg/mL, respectively. Percent recovery of mannitol from spiked urine samples ranged from 90.0% to 125.0%. Percent recovery of lactulose from spiked urine samples ranged from 77.5% to 123.0%. All values below the detection limit were reported as less than the detection limits of mannitol and the detection limits of lactulose. The intra-assay CVs for LM were 6.8% and 1.2%, respectively. To ensure reproducibility, batches of the same samples (*n* = 14) were analyzed in duplicate at Wagner College and the Mayo Clinic. The inter-assay CV between the 2 laboratories was 5.2% for lactulose and 3.8% for mannitol. Cross-check analyses were conducted periodically throughout the sample analysis process in collaboration with the Mayo Clinic.

#### Outcomes: child neurodevelopment measurements

The child neurodevelopment measurements of the parent trials were previously described [38,40]. Briefly, the MacArthur-Bates Communicative Developmental Inventories measures language development evaluated by parental reports [38,41]. This metric has been validated for and was used only in Bangladesh at years 1 and 2 [38].

The WHO motor milestones tool was assessed at year 1 in both Kenya and Bangladesh to measure 6 gross motor milestones: sitting without support, hands-and-knees crawling, standing with assistance, walking with assistance, standing alone, and walking alone [38,40,42]. It consists of a direct at-home assessment and a parental report. Analyses reporting “sum total” tallied the total number of milestones children were reported to have attained by follow-up. There were high levels of nonattainment for milestones 1 and 3, despite the assumption that children logically progress through each milestone. Therefore, “milestones 2,4,5,6” tallied the sitting without support, standing with assistance, walking with assistance, standing alone, and walking alone milestones attained by follow-up.

The Extended Ages and Stages Questionnaire (EASQ) is a parental-report measure of child development, which includes 3 EASQ domains, child communication, gross motor, and personal social [38,40,43,44]. When this tool was adapted for use in Bangladesh by ICDDR,B, ∼25% of the items included direct observation to assess behaviors that may not be reliably reported by parents [38]. These direct assessment items were specifically selected to address behaviors that are less observable in the home environment, such as pointing at pictures in a book, naming body parts, or performing simple motor tasks such as kicking a ball or copying gestures. The inclusion of direct observation aims to improve the accuracy and comprehensiveness of the developmental assessment. This adaptation was outlined in the Supplementary Appendix of the primary WASH Benefits child development paper, where the full list of the direct assessment items can be found [38]. For context, the standard Ages and Stages Questionnaire (ASQ-3) does not require direct assessment but does allow for it as an optional addition. In contrast, the modified EASQ ensures a more reliable evaluation by including direct observation for ∼25% of items, particularly in settings where parental reports may be less accurate. The tool was also adapted for use in Kenya [40]. The domain-specific and the cumulative global EASQ scores were age standardized to a reference group (the control arm in Bangladesh and active control arm in Kenya) and included in the outcomes. EASQ was measured at year 2 in Bangladesh and Kenya.

### Statistical analysis

A preregistered statistical analysis plan can be found on Open Science Framework (https://osf.io/xahgw/). We hypothesized that higher concentrations of each of the EED biomarkers—where increased concentrations of alpha-1 antitrypsin, lactulose, and mannitol indicate greater intestinal permeability, elevated myeloperoxidase and neopterin reflect higher inflammation, and increased REG1B signifies enhanced intestinal repair—would be negatively associated with child neurodevelopment. Analyses were conducted using R statistical software, 4.1.0. Because all EED biomarker distributions were right-skewed, we natural log-transformed them. To assess the correlation between LM concentrations, we calculated Pearson correlation coefficients at the follow-up 1 and follow-up 2 timepoints in Bangladesh and Kenya.

We conducted analyses of each exposure–outcome pair at subsequent timepoints (e.g., alpha-1 antitrypsin measured at follow-up 1 was analyzed for associations with the MacArthur-Bates Communicative Development Inventories at years 1 and 2). Estimated mean differences were calculated using natural smoothing splines and generalized additive models. To avoid assuming linear relationships a priori and missing any nonmonotonicity and curvilinear relationships, we fit natural splines for each exposure–outcome pair (Supplemental Appendix A). For interpretability across the large number of exposures and outcomes, we compared the 75th with the 25th percentile of each exposure in the preregistered statistical analysis plan, which conceptually yields a contrast between “higher” compared with “lower” biomarker levels comparable across exposure–outcome pairs. Hazard ratios relating EED markers to attainment of individual WHO motor milestones were calculated using complementary log–log link and baseline hazard fit with monotonic cubic splines, as done previously [38,40]. Both mean differences and hazard ratios compared the 75th with the 25th percentiles to summarize general direction and magnitude of the association. We used a “sum total” of WHO motor milestones and a sum score of “milestones 2,4,5,6” at year 1 primarily as a pragmatic snapshot of overall gross-motor achievement. Summing all attained milestones (rather than excluding children who “skipped” or achieved them out of sequence) reduces dimensionality while still reflecting each child’s relative progress. To respect the inherently ordinal nature of the WHO motor milestones, we also examined each one using hazard-ratio models that approximate “time to attainment.” These models draw on parental recall plus in-person observation at discrete follow-ups, providing a partial age-of-attainment measure—albeit with limited resolution due to only 2 major visits. We, therefore, chose to present both a summed-milestone outcome (for a single measure of progress) and milestone-specific hazard ratios (for a more event-based approach).

In this analysis, we assessed the strength of associations between individual EED markers and the consistency of the direction of these associations among related biomarker groups (e.g., intestinal permeability markers: lactulose, mannitol, and alpha-1-antitrypsin) using 2 complementary approaches: one focusing on the false discovery rate (FDR) and the other on the coherence of associations across multiple measures within the same exposure–outcome domain (e.g., intestinal permeability markers and WHO motor milestones). The FDR approach aims to adjust for the number of repeated tests to evaluate the likelihood that individual results arise from random chance. FDR correction was grouped by hypothesis, which was determined by the time point the outcome was measured at, as prespecified in the registered analysis plan (https://osf.io/73xcz/). *P* values for models adjusted for covariates were reported, along with their corresponding FDR-corrected *P* value corrected using the Benjamini-Hochberg method to account for multiple testing [[45], [46], [47]]. A *P* value < 0.05 was considered statistically significant. However, in interpreting these results, we also considered whether multiple measures of a similar exposure–outcome domain (e.g., intestinal permeability markers and WHO motor milestones) displayed a consistent pattern of associations. For example, if a specific exposure–outcome domain (e.g., intestinal inflammation and EASQ combined scores) showed mixed directional associations (i.e., some positive and some negative correlations), whereas individual measures indicated statistically significant results, we interpreted these individual associations as potentially spurious (e.g., false positives or false negatives), attributable to repeated testing. Conversely, when we identified a consistent directional pattern across multiple measures within the same exposure–outcome domain—such as consistent negative associations between intestinal permeability markers and WHO motor milestones—but only 1 individual association (e.g., mannitol and hands-and-knees crawling milestone) remained significant after FDR correction, we interpreted these findings as indicative of a potentially true relationship between the exposure and outcome domains. This suggests that, despite the lack of significance in most individual associations, the overarching pattern may still reflect meaningful underlying relationships. To evaluate the robustness of individual associations while accounting for the effects of repeated testing, we applied both the Benjamini-Hochberg method for FDR correction and our qualitative assessment of directional consistency. This dual approach allowed for a more nuanced understanding of the associations between EED markers and child development outcomes.

The full covariate list was prespecified on Open Science Framework in the preregistered statistical analysis plan (https://osf.io/73xcz/) and included the following covariates: birth order, maternal age, height, and education, food insecurity, household crowding, access to drinking water, household assets, prior growth, treatment arm, month of assessment, assessment time, and maternal depression, stress, and lifetime exposure to any type of intimate partner violence (further details in Supplemental Table 1). All adjusted analyses controlled for child age, sex, and other covariates found to be related to the outcome in bivariate analyses (*P* < 0.2). The complete list of prespecified covariates underwent a prescreening and selection process, where we assessed potential confounding by using the likelihood ratio test to evaluate the association between each outcome and covariate. Covariates with a *P* value < 0.2 were included in the analysis, and covariates with little variation in the study population (prevalence < 5%) were excluded. Additionally, continuous variables with > 10% missingness were categorized to reduce the number of observations dropped due to missingness in the adjusted analysis. Models included random effects at the cluster level to account for repeated measures. Additionally, the treatment group was included as a covariate in the model to control for its potential confounding effects on the outcome.

In post hoc sensitivity analyses, we repeated analyses using linear and Cox regressions [without spline terms, and with bootstrapped confounder selection and confidence intervals (CIs)] (Supplemental Appendix B).

Post hoc sensitivity analyses were also performed in both countries to explore lactation and diarrhea as potential confounders. We performed *t* tests comparing the mean EED biomarker concentrations between children who were exclusively fed human milk and those who were not exclusively fed human milk. Sensitivity analyses were subsequently performed comparing the results of adjusted models with the exclusive human milk feeding variable included, to models without the variable, using the variance inflation factor to drop variables that were found to be collinear with exclusive human milk feeding. In post hoc sensitivity analyses, diarrhea was included as an adjustment variable. Because diarrhea was measured concurrently or before the EED measurements, these sensitivity analyses controlled for diarrhea as a confounder (Supplemental Appendix C).

In Bangladesh, the prevalence of *Ascaris lumbricoides*, hookworm, and *Trichuris trichiura* at age 14 mo was previously reported [36]. In Bangladesh, we conducted a post hoc subgroup analysis stratified by soil-transmitted helminth infection because helminth infection could modify the associations between EED and child development. Similar analyses were not conducted in Kenya because soil-transmitted helminths were not assessed at age 17 mo. In Bangladesh and Kenya, soil-transmitted helminths were previously assessed at year 2, with prevalence for *A. lumbricoides*, hookworm, and *T. trichiura* reported in each setting [34,39]. However, effect measure modification analyses were not performed for year 2 in either country, as the soil-transmitted helminth assessments took place after the child development measures, thereby limiting the analyses due to temporal ordering.

## Results

### Enrollment characteristics

The parent trials enrolled 5551 and 8246 pregnant females and their children in Bangladesh and Kenya, respectively. A total of 2646 children with EED and neurodevelopment measurements were included in this analysis, 1374 in Bangladesh and 1272 in Kenya (Supplemental Figures 1 and 2).

Child, mother, and household characteristics are summarized in TABLE 1, TABLE 2. For EED biomarker measurements, follow-up 1 measurements occurred when children were at a median age of 3 mo (IQR: 1–4 mo) in Bangladesh and 6 (IQR: 4–6 mo) mo in Kenya (TABLE 1, TABLE 2). Follow-up 2 is defined as the measurement when the median child age was 14 (IQR: 13–15 mo) mo in Bangladesh and 17 (IQR: 15–18 mo) mo in Kenya. In Bangladesh and Kenya, the concentrations of LM were strongly and significantly correlated at both time points (Bangladesh follow-up 1: *r* = 0.85 and follow-up 2: *r* = 0.74; Kenya follow-up 1: *r* = 0.85 and follow-up 2: *r* = 0.84; *P* < 0.001). For child development measures, follow-up 2 (year 1) measurements occurred when the children were at median age of 11 (IQR: 10–13 mo) mo in Bangladesh and 12 (IQR: 11–13 mo) mo in Kenya. Follow-up 3 (year 2) measurements occurred when the children were at a median age of 26 (IQR: 24–27 mo) mo in Bangladesh and 25 (IQR: 23–26 mo) mo in Kenya. Household enrollment and child characteristics of the subsample were similar to those from the parent trial (TABLE 1, TABLE 2; Supplemental Tables 2 and 3). Household enrollment and child characteristics were generally balanced between individuals who had child development outcomes at year 1 and those who were lost to follow-up at year 2 (Supplemental Tables 2 and 3). In Bangladesh, a higher proportion of children lost to follow-up at year 1 were from severely food-insecure households; however, this observation was likely due to chance and the small sample size of children lost to follow-up (*N* = 17).

**TABLE 1:** Characteristics of participants in Bangladesh.

Characteristic	*N* = 1599^1^
Child	
Female (%)	798 (49.91)
Length-for-age *Z* score at age 3 (mo)	–1.28 (–1.99, –0.57)
Weight-for-age *Z* score at age 3 (mo)	–1.19 (–1.84, –0.56)
Length-for-age *Z* score at age 14 (mo)	–1.39 (–2.07, –0.76)
Weight-for-age *Z* score at age 14 (mo)	–1.31 (–2.00, –0.63)
Caregiver-reported diarrhea 7-d recall at age 3 (mo)	211 (14.05%)
Caregiver-reported diarrhea 7-d recall at age 14 (mo)	99 (7.09%)
Age at EED biomarker measurement, follow-up 1 (mo)	2.75 (1.64, 4.07)
Age at EED biomarker measurement, follow-up 2 (mo)	14.03 (12.59, 15.34)
Age at child development measurement, follow-up 2 (mo)	11.41 (10.20, 12.66)
Age at child development measurement, follow-up 3 (mo)	25.61 (24.36, 26.82)
Mother	
Age (y)	24.00 (20.00, 27.00)
Height (cm)	150.40 (146.80, 154.15)
Depressive symptoms (CESD-R score) at year 1	22 (8, 47)
Depressive symptoms (CESD-R score) at year 2	24.00 (7.00, 54.00)
Maternal Perceived Stress Scale (PSS) at year 2	12.00 (7.00, 32.00)
Education completed	
No education	224 (14.01%)
Primary (1–5 y)	453 (28.33%)
Secondary ( >5 y)	922 (57.66%)
Household	
Household food insecurity^2^	
Mildly food insecure	132 (8.26%)
Moderately food insecure	284 (17.76%)
Severely food insecure	55 (3.44%)
Food secure	1128 (70.54%)

Abbreviations: EED, environmental enteric dysfunction; Q1, first quartile; Q3, third quartile; CESD-R, Center for Epidemiologic Studies Depression Scale Revised.

1 *n* (%); median (Q1, Q3).

2 Any level of food insecurity assessed using the Household Food Insecurity Access Scale.

**TABLE 2:** Characteristics of participants in Kenya.

Characteristic	*N* = 1703^1^
Child	
Female (%)	828 (48.62)
Length-for-age *Z* score at age 6 (mo)	–0.77 (–1.46, –0.07)
Weight-for-age *Z* score at age 6 (mo)	–0.25 (–0.99, 0.41)
Length-for-age *Z* score at age 17 (mo)	–1.27 (–1.99, –0.55)
Weight-for-age *Z* score at age 17 (mo)	–0.64 (–1.29, 0.03)
Caregiver-reported diarrhea 7-d recall at age 6 (mo)	82 (41.21%)
Caregiver-reported diarrhea 7-d recall at age 17 (mo)	477 (30.68%)
Age at EED biomarker measurement, follow-up 1 (mo)	5.54 (4.16, 6.79)
Age at EED biomarker measurement, follow-up 2 (mo)	16.66 (15.08, 18.10)
Age at child development measurement, follow-up 2 (mo)	12.36 (10.98, 13.67)
Age at child development measurement, follow-up 3 (mo)	24.43 (23.11, 25.67)
Mother	
Age (y)	25.52 (21.38, 30.01)
Height (cm)	160.50 (156.40, 164.40)
Depressive symptoms (PHQ) at year 2	5.00 (2.00, 8.00)
Maternal Perceived Stress Scale (PSS) at year 2	18.00 (13.00, 23.00)
Education completed	
Primary	403 (23.66%)
Incomplete primary	913 (53.61%)
Any secondary	385 (22.61%)
Missing	2 (0.12%)
Household	
Prevalence of moderate-to-severe household hunger^2^	172 (10.10%)

Abbreviations: EED, environmental enteric dysfunction; Q1, first quartile; Q3, third quartile; PHQ, Patient Health Questionnaire

1 *n* (%); median (Q1, Q3).

2 Moderate-to-severe hunger defined using the Household Hunger Scale.

In Bangladesh, the caregiver reported that 7–d recall of diarrhea was 14% at follow-up 1, and in Kenya, it was 41% at follow-up 1 (TABLE 1, TABLE 2). The prevalence of moderate-to-severe food insecurity was 21% in Bangladesh, and the prevalence of moderate-to-severe household hunger was 10% in Kenya. In Bangladesh, 58% of the mothers completed secondary education, whereas 23% of the mothers in Kenya completed secondary education.

### EED biomarkers and neurodevelopment in Bangladesh

**FIGURE 2:**
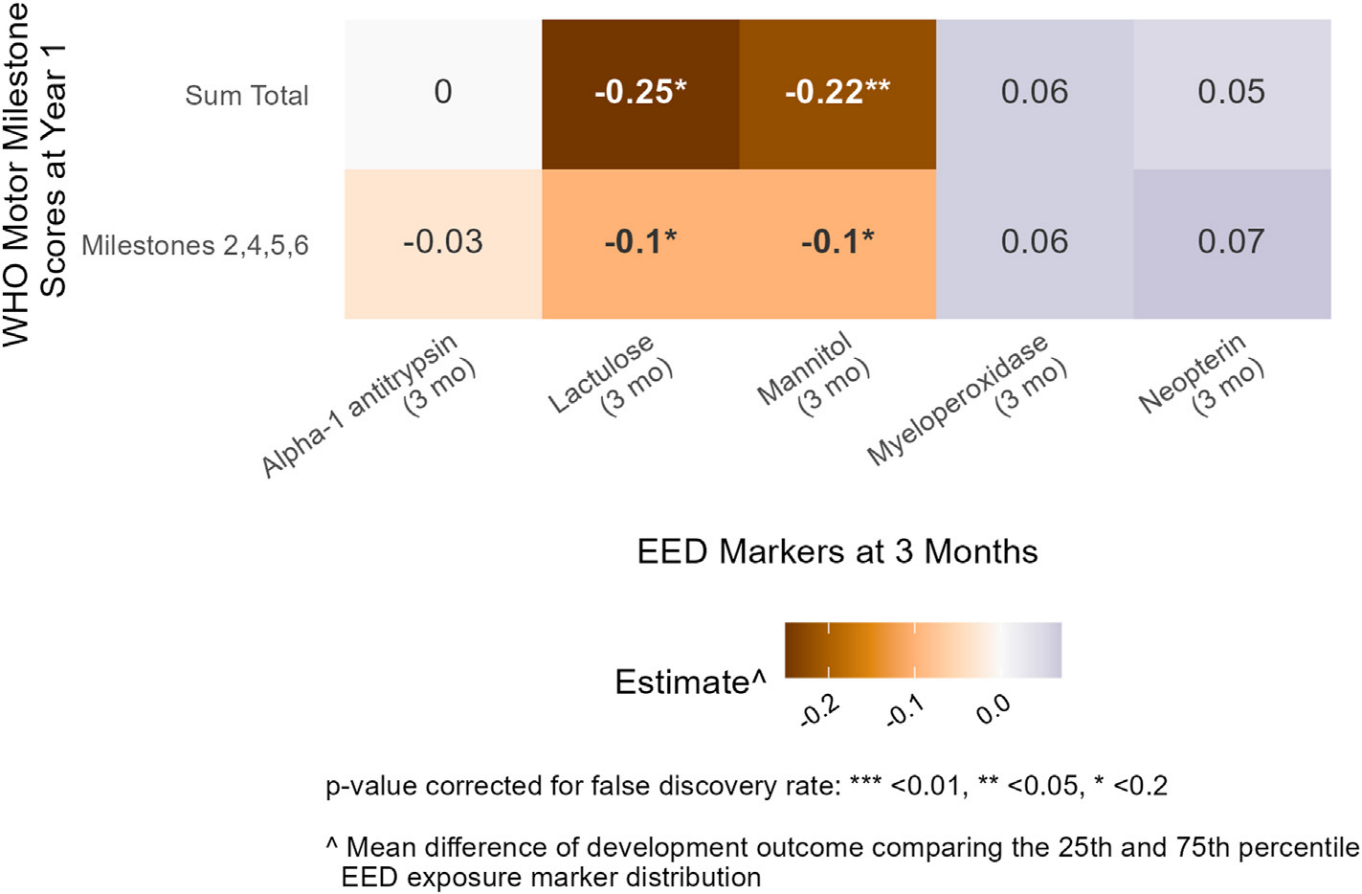
Adjusted mean differences in WHO motor milestone scores at 1 year between the 25th and 75th percentile of environmental enteric dysfunction (EED) biomarkers at 3 mo (follow-up 1) in Bangladesh. Adjusted for prespecified and prescreened covariates: child sex, child age, child birth order, mother’s age, mother’s height, mother’s education, household food security, number of children aged < 18 y in the household, number of people living in the compound, distance (in minutes) to the primary water source, household materials (floor, roof, and walls), asset-based household wealth (electricity, wardrobe, table, chair or bench, watch or clock, khat, chouki, working radio, working black/white or color television, refrigerator, bicycle, motorcycle, sewing machine, mobile phone, land phone, number of cows, number of goats, and number of chickens), treatment arm, prior length-for-age *Z* score and weight-for-age *Z* score, month of measurement, mother’s depressive symptoms, and mother’s exposure to intimate partner violence (IPV) during lifetime.

At follow-up 1 in Bangladesh (age 3 mo), mannitol concentrations were negatively associated with the subsequent “sum total” of all WHO motor milestones (adjusted difference in the “sum total” of all WHO motor milestones between the 25th and 75th percentile of mannitol distribution: –0.22; 95% CI: –0.36, –0.08) (Figure 2, Supplemental Table 4). These results remained significant after FDR correction (*P* = 0.03). The gut permeability markers were consistent in the magnitude and direction of associations but the lactulose results were not significant after FDR correction (Figure 2, Supplemental Table 4). The associations between mannitol and lactulose and the subsequent “milestones 2,4,5,6” were consistent with the “sum total” of all the WHO motor milestones; however, these associations were somewhat attenuated (Figure 2, Supplemental Table 4).

**FIGURE 3:**
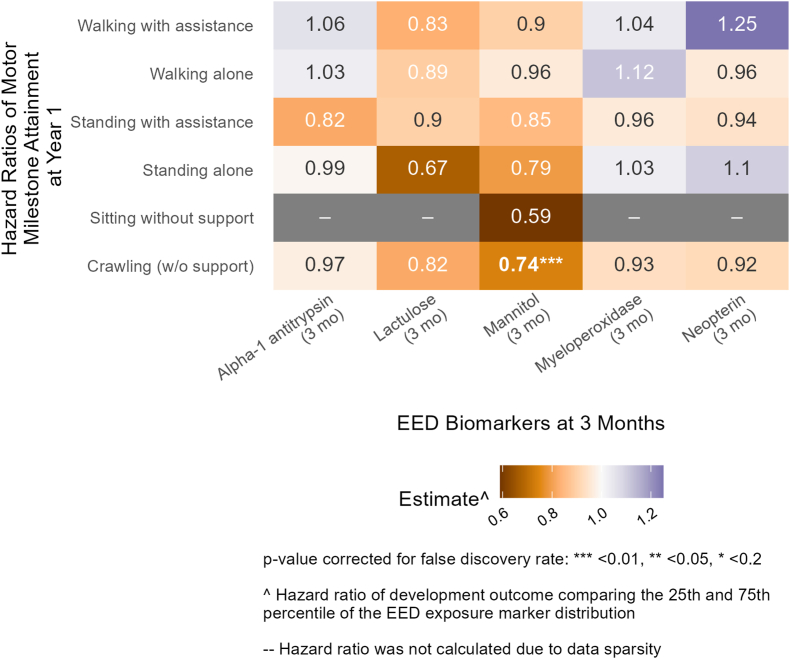
Relative rate of WHO motor milestone attainment at 1 y between the 25th and 75th percentile of environmental enteric dysfunction (EED) biomarkers at 3 mo (follow-up 1) in Bangladesh. Adjusted for prespecified and prescreened covariates: child sex, child age, child birth order, mother’s age, mother’s height, mother’s education, household food security, number of children aged < 18 y in the household, number of people living in the compound, distance (in min) to the primary water source, household materials (floor, roof, and walls), asset-based household wealth (electricity, wardrobe, table, chair or bench, watch or clock, khat, chouki, working radio, working black/white or color television, refrigerator, bicycle, motorcycle, sewing machine, mobile phone, land phone, number of cows, number of goats, and number of chickens), treatment arm, prior length-for-age *Z* score and weight-for-age *Z* score, month of measurement, mother’s depressive symptoms, and mother’s exposure to intimate partner violence (IPV) during lifetime.

LM concentrations at follow-up 1 (age 3 mo) in Bangladesh had consistent negative associations with attainment of several WHO motor milestones at age 1 y. A hazard ratio of 0.67 (95% CI: 0.47, 0.96) for the standing-alone milestone indicates that children in the 75th (high) percentile of lactulose concentration attained the milestone 0.67 times as much as children in the 25th (low) percentile (Figure 3, Supplemental Table 5). Therefore, higher lactulose concentrations were associated with a lower rate of attainment of the standing-alone milestone. Similarly, higher mannitol concentrations were associated with lower rates of attainment for the hands-and-knees crawling milestone (hazard ratio: 0.74; 95% CI: 0.64, 0.86), the standing with assistance milestone (hazard ratio: 0.85; 95% CI: 0.72, 1), and the standing-alone milestone (hazard ratio: 0.79; 95% CI: 0.63, 0.99). Alpha-1 antitrypsin, an intestinal permeability marker, at follow-up 1 (age 3 mo) also had a negative association with the standing with assistance milestone (hazard ratio: 0.82; 95% CI: 0.66, 1.02). The association between mannitol and the hands-and-knees crawling milestone remained significant after FDR correction (*P* < 0.001).

**FIGURE 4:**
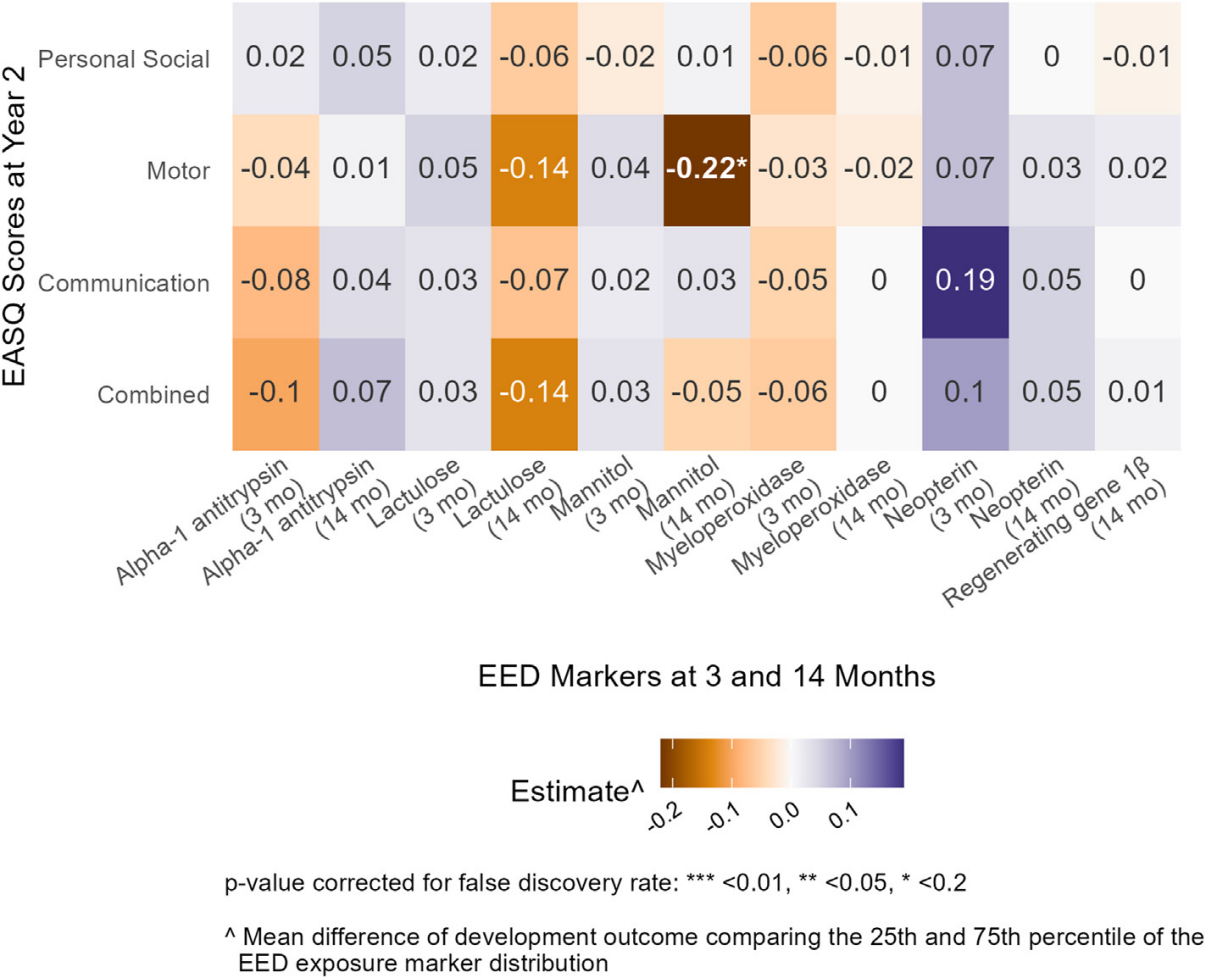
Adjusted mean differences in scores on the communication, gross motor, personal social, and combined scales of the Extended Ages and Stages Questionnaire (EASQ) at 2 y between the 25th and 75th percentile of environmental enteric dysfunction (EED) biomarkers at 3 and 14 mo (follow-ups 1 and 2) in Bangladesh. Adjusted for prespecified and prescreened covariates: child sex, child age, child birth order, mother’s age, mother’s height, mother’s education, household food security, number of children aged < 18 y in the household, number of people living in the compound, distance (in min) to the primary water source, household materials (floor, roof, and walls), asset-based household wealth (electricity, wardrobe, table, chair or bench, watch or clock, khat, chouki, working radio, working black/white or color television, refrigerator, bicycle, motorcycle, sewing machine, mobile phone, land phone, number of cows, number of goats, and number of chickens), treatment arm, prior length-for-age *Z* score and weight-for-age *Z* score, month of measurement, mother’s perceived stress, mother’s depressive symptoms, and mother’s exposure to intimate partner violence (IPV) during lifetime.

LM concentrations at follow-up 2 (age 1 y) were negatively associated with the gross motor domain of EASQ at year 2 {[adjusted differences: –0.14 (–0.26, –0.01) and –0.22 (–0.36, –0.07)], respectively; Figure 4, Supplemental Table 4}; however, the results for LM at follow-up 2 were not significant after FDR correction.

In Bangladesh, self-reported exclusive lactation prevalence at follow-up 1 (age 3 mo) was 36.6%. In post hoc analyses, we explored the possibility of exclusive lactation as a confounder by performing *t* tests. *T* tests comparing mean LM concentrations between parents reporting having exclusively fed their children human milk and those who did not exclusively feed their children human milk were found to be significantly different (*P* < 0.01). In sensitivity analyses, forced inclusion of the exclusive lactation variable as an adjustment covariate in the generalized additive models yielded similar point estimates as the prespecified main analyses. For example, between the 25th and 75th percentiles of lactulose concentrations at age 3 mo, there was a difference in WHO “sum total” scores of –0.22 (–0.47, 0.03; Supplemental Table 6) adjusting for exclusive lactation and a difference of –0.25 (–0.50, –0.01) without adjustment. Similarly, between the 25th and 75th percentiles of mannitol concentrations at age 3 mo, there was a difference of WHO “sum total” scores –0.23 (−0.38, −0.07) with adjustment for the exclusive lactation variable and –0.22 (–0.36, –0.08) without adjustment (Supplemental Table 6). In the Bangladesh EED substudy, the caregiver-reported 7–d diarrhea prevalence was 7.5% at age 14 mo. In post hoc sensitivity analyses, the forced inclusion of diarrhea as an adjustment variable in the models produced similar point estimates as the prespecified main analyses (Supplemental Appendix C).

In Bangladesh, the soil-transmitted helminth prevalence at age 14 mo was <5% [36]. In post hoc analyses, there was no evidence of effect measure modification by soil-transmitted helminth infection at age 14 mo. The soil-transmitted helminth prevalence at year 2 was 36.8% for *A. lumbricoides*, 9.2% for hookworm, and 7.5% for *T. trichiura* [34]. At year 2, the measurement of soil-transmitted helminths was conducted after assessing the child development measures; thus, year 2 effect measure modification analyses were not performed due to timing limitations.

### EED biomarkers and neurodevelopment in Kenya

**FIGURE 5:**
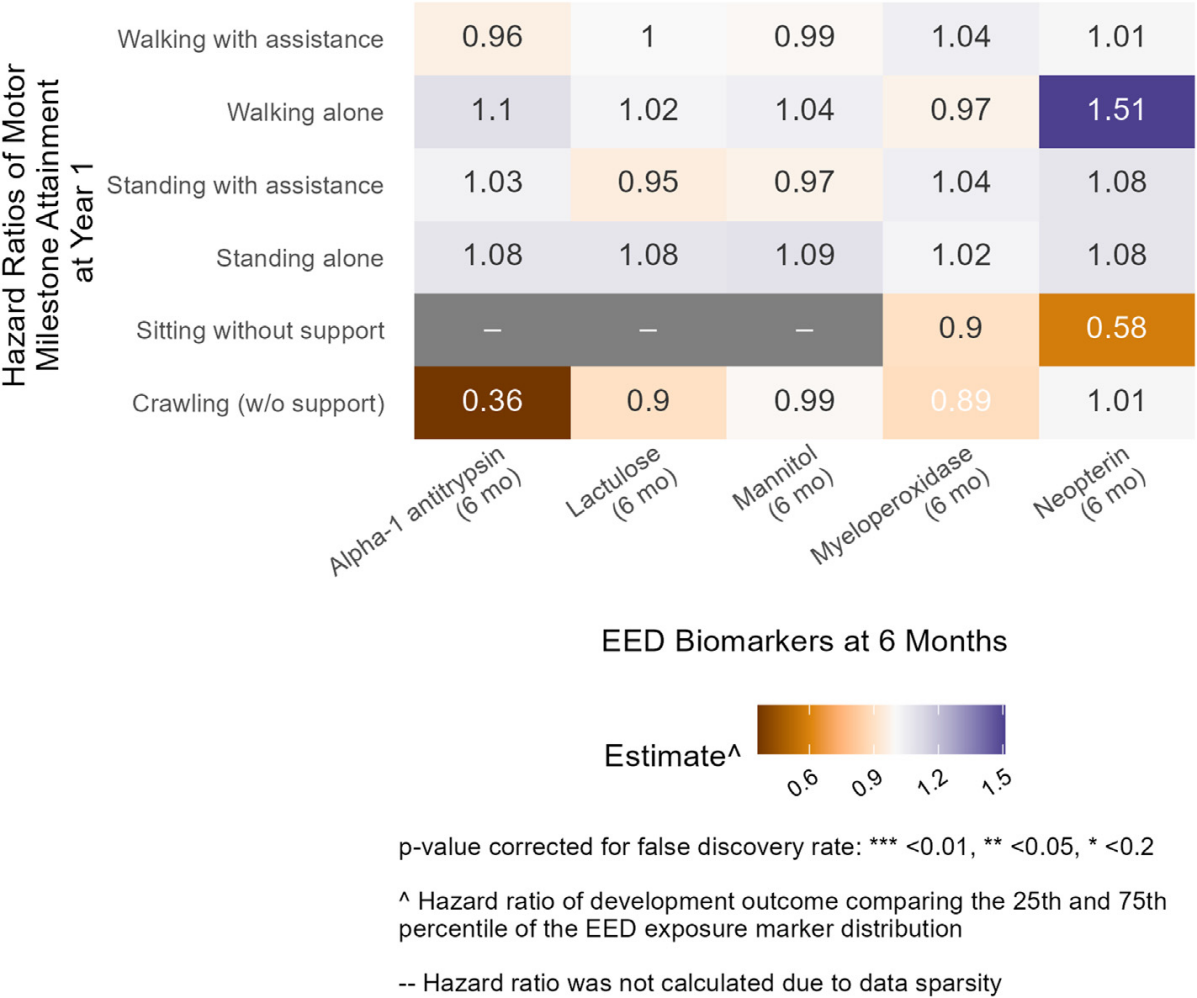
Relative rate of WHO motor milestone attainment at 1 y between the 25th and 75th percentile of environmental enteric dysfunction (EED) biomarkers at 6 mo (follow-up 1) in Kenya. Adjusted for prespecified and prescreened covariates: child sex, child age, child birth order, mother’s age, mother’s height, mother’s education, Household Hunger Scale, number of children aged < 18 y in the household, number of people living in the compound, distance (in min) to the primary water source, household materials (floor, roof), asset-based household wealth (electricity, clock, working radio, working black/white or color television, bicycle, motorcycle, sewing machine, mobile phone, land phone, stove, number of cows, number of goats, number of dogs, and number of poultry), treatment arm, prior length-for-age *Z* score and weight-for-age *Z* score, and month of measurement.

**FIGURE 6:**
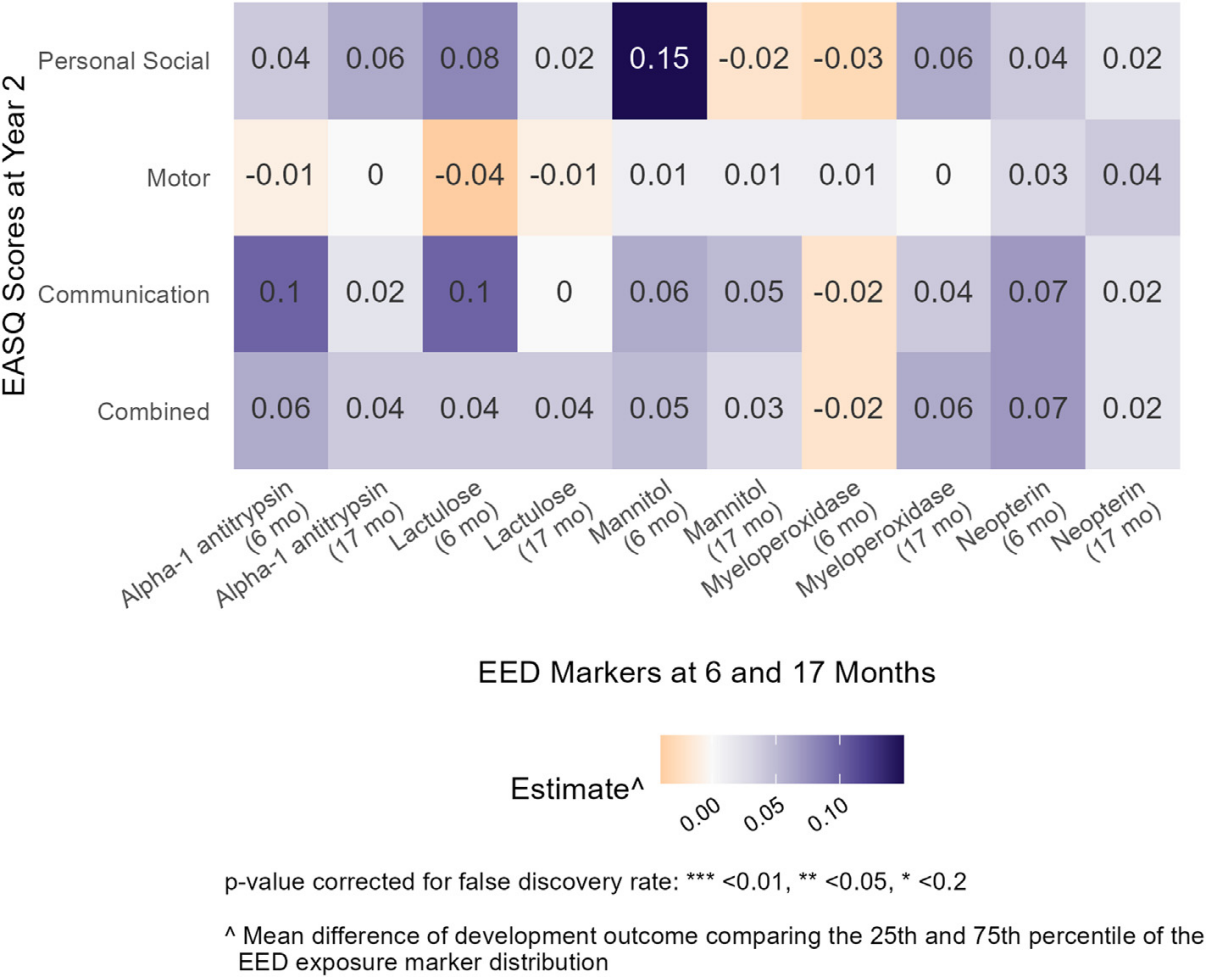
Adjusted differences in scores on the communication, gross motor, personal social, and combined scales of the Extended Ages and Stages Questionnaire (EASQ) at 2 y between the 25th and 75th percentiles of environmental enteric dysfunction (EED) biomarkers at 6 and 17 mo (follow-ups 1 and 2) in Kenya. Adjusted for prespecified and prescreened covariates: child sex, child age, child birth order, mother’s age, mother’s height, mother’s education, Household Hunger Scale, number of children aged < 18 y in the household, number of people living in the compound, distance (in minutes) to the primary water source, household materials (floor, roof), asset-based household wealth (electricity, clock, working radio, working black/white or color television, bicycle, motorcycle, sewing machine, mobile phone, land phone, stove, number of cows, number of goats, number of dogs, and number of poultry), treatment arm, prior length-for-age *Z* score and weight-for-age *Z* score, month of measurement, mother’s perceived stress, and mother’s depressive symptoms.

In Kenya, we observed predominantly weak associations, indicated by larger *P* values and a lack of consistency in direction between EED biomarkers and WHO motor milestones. At follow-up 1 (age 6 mo), higher measures of alpha-1 antitrypsin were associated with a lower rate of attaining the hands-and-knees crawling WHO motor milestone at year 1 (Figure 5, Supplemental Table 7). However, the directionality of effects was inconsistent, and the magnitudes were small for LM (the other markers of intestinal permeability) and for other WHO motor milestones. Children with higher measures of intestinal permeability and inflammation generally exhibited better EASQ outcomes at year 2, although the associations were very weak. At follow-up 1 (age 6 mo), mannitol concentrations were positively associated with the personal social score of EASQ at year 2 (adjusted difference: 0.15; 95% CI: 0.04, 0.25) (Figure 6, Supplemental Table 8). None of the results in Kenya for inflammation and permeability were significant after FDR correction.

There was a 12.4% prevalence of exclusive lactation in Kenya at follow-up 1. Concentrations of alpha-1 antitrypsin and lactulose were observed to be significantly different among children of parents who reported having exclusively lactated and those who did not. Sensitivity analyses, forcing the inclusion of the exclusive lactation variable as an adjustment covariate in the generalized additive models, yielded estimates with similar magnitude and direction as the prespecified main analyses (Supplemental Table 9). In Kenya, the caregiver-reported 7–d diarrhea prevalence was 31% at age 17 mo. In post hoc sensitivity analyses, including diarrhea as a forced adjustment variable in the models resulted in point estimates that were similar to those from the prespecified main analyses (Supplemental Appendix C). In Kenya, soil-transmitted helminths were not measured at age 17 mo, but at year 2, the soil-transmitted helminth prevalence was 23% for *A*. *lumbricoides*, 2% for hookworm, and 1% for *T*. *trichiura* [39]. As in the case of Bangladesh, the soil-transmitted helminth assessment at year 2 occurred after the child development assessment. Consequently, effect measure modification analyses for year 2 were not performed due to the same limitations.

### Post hoc sensitivity analyses confirming the robustness of the findings

We fit natural splines for each exposure–outcome pair that demonstrated that the 25th–75th comparisons for our primary findings did not have any nonmonotonic or strongly curvilinear relationships that were obscured by the a priori chosen comparisons (Supplemental Appendix A). Additionally, repeated analyses employing both linear and Cox regressions (without spline terms, and incorporating bootstrapped confounder selection and CIs) yielded consistent results (Supplemental Appendix B), further confirming the robustness of the findings and demonstrating that the primary conclusions are not dependent on any particular modeling approach.

## Discussion

Measures of intestinal permeability were associated with subsequent child neurodevelopment measurements in Bangladesh. Elevated intestinal permeability, as indicated by higher concentrations of lactulose, mannitol, and alpha-1 antitrypsin, was associated with worse subsequent child neurodevelopment outcomes in Bangladesh. The associations between intestinal permeability and neurodevelopmental outcomes were generally large in magnitude and consistently negative across WHO motor milestones at year 1 and the gross motor domain of the EASQ at year 2. Intestinal inflammation was not associated with neurodevelopment in Bangladesh. In Kenya, we observed generally weak associations characterized by a lack of consistency in direction between EED markers and child neurodevelopment outcomes.

In Bangladesh, elevated LM concentrations (both indicating elevated intestinal permeability [24]) at follow-up 1 (age 3 mo) were associated with delayed rates of attainment of WHO motor milestones at age 1 y. The higher intestinal permeability enables bacteria and bacterial products to translocate into systemic circulation [48,49]. This translocation can lead to immune activation, potentially triggering systemic inflammation, which, in turn, may divert much-needed resources away from child neurodevelopment [48,50]. A study in Zambia reported that elevated alpha-1-acid glycoprotein, a marker of systemic inflammation, and intestinal fatty acid-binding protein (I-FABP), a marker of gut barrier dysfunction, were associated with poor child development outcomes [31]. Furthermore, Jiang et al. [27] found that proinflammatory cytokines, IL-6 and IL-1β, were associated with poorer child development outcomes in Bangladesh. Elevated concentrations of IL-6 have previously been linked to impaired cognition [[51], [52], [53], [54], [55], [56]]. Similarly, in our study, higher concentrations of intestinal permeability markers were linked to impaired motor skill development, potentially mediated by a proinflammatory immune response to pathogens.

In Kenya, we observed generally weak associations, with inconsistent directionality between EED markers and WHO motor milestones at year 1. Children with higher levels of intestinal permeability and inflammation generally had better EASQ outcomes at year 2, although the associations were weak. A possible explanation is that the pathogen profile of each country elicited different immune responses, which in turn impacted child development differently. The prevalence of soil-transmitted helminth infections was <5% at age 14 mo in Bangladesh [36], and helminths did not modify the effect of EED biomarkers on child development measures. In Kenya, helminths were not assessed at age 17 mo. Although the prevalence of soil-transmitted helminth infections was higher in Bangladesh than in Kenya at year 2, effect measure modification analyses could not be conducted due to limitations in the temporal ordering of the potential modifiers and outcomes. An immunoregulatory response, such as the one associated with IL-4 in a T helper 2 (Th2) response, might have led to improved child neurodevelopment despite increased exposure to microbial translocation [27,57,58]. Jiang et al. [27] observed opposing effects of IL-4 and IL-6 in their Bangladeshi cohort, noting that increased IL-4 concentrations have been associated with better cognition in animal models [59]. A study in Tanzania also observed positive associations between EED markers and cognitive scores and suggested that this was due to a more robust immune response [28].

It is possible the different geographic settings elicited different immune responses. How children’s immune systems respond to pathogens is likely influenced by the environmental pathogen and microbiome profile of each country or region [60]. Genetic factors could also play a role, exemplified by the rare C variant (instead of G) in the promoter gene of IL-6, which increases IL-6 synthesis and has been linked to adverse child development in children born prematurely [61]. Lastly, it could be a combination of environment, epigenetics, and genetics, where the dynamic interaction of the environmental exposures and a child’s epigenome and genome leads to variations in immune responses and subsequently, variations in child neurodevelopment.

The overall intercountry differences between EED biomarkers and child development scores could also be due to differences in diarrhea prevalence [32,33], nutritional status, parental education, or other factors. In this substudy, the 7-d diarrhea prevalence was 31% at age 17 mo in Kenya and 7.5% at age 14 mo in Bangladesh. However, post hoc sensitivity analyses, where diarrhea was included as an adjustment variable in the models, resulted in similar results to those from the prespecified main analyses. We adjusted for child nutritional status [length-for-age *Z* score and weight-for-age *Z* score] and maternal education in our models (Supplemental Table 1).

This study had several strengths. The analyses were prespecified and registered on Open Science Framework (https://osf.io/xahgw/). The study had the temporal ordering necessary to examine relationships between EED and child neurodevelopment measurements. Conducting this study in 2 epidemiologic settings, Bangladesh and Kenya, increased the generalizability of the findings. This study was also limited. Although we can hypothesize potential factors, such as diarrhea prevalence or lactation practices, that may explain the observed inconsistencies in the associations, we were unable to fully account for these variables in these analyses, as they were measured at the same time points as the EED exposure. However, the results of the sensitivity analyses indicate minimal potential bias from diarrhea or exclusive lactation. Another potential limitation is, despite extensive training for testers [38], the parental-report components for some of the child development measures could introduce reporting errors or bias. Due to the observational nature of this study, unmeasured confounding, such as environmental conditions or familial risk factors, may be present.

The large number of comparisons at different time points increases the potential for type 1 errors. To mitigate this risk, we first assessed not only the consistency of the direction (positive compared with negative) of point estimates across different exposure–outcome domains and evaluated the statistical significance of each contrast, but we also applied the Benjamini-Hochberg procedure for FDR correction. However, the FDR correction may be overly conservative and reduce statistical power, particularly when exposure measures (i.e., EED markers) are highly correlated and small to modest effect sizes are generally expected. We also recognize that categorizing continuous variables with >10% missingness coarsens the data, but this analytical choice was a decision to avoid excessive loss of observations in adjusted models. Our approach was specified in the preregistered analysis plan (https://osf.io/xahgw/). This study was limited by its exclusion of some important EED domains, including microbial translocation and microbiome dysfunction [62]. Future research should incorporate these additional domains to provide a more comprehensive understanding of the links between EED and neurodevelopment.

We found that elevated intestinal permeability was associated with worse subsequent child neurodevelopment measurements in the Bangladesh cohort. However, this association was not observed in the Kenyan cohort. Similar to a previous study in Tanzania, we observed very weak positive associations between intestinal permeability and subsequent child neurodevelopment in Kenya [28]. Associations between EED and child neurodevelopment may vary across different populations, suggesting that future research should focus on determining the environmental factors (e.g., pathogens and chemical exposures) and biological mechanisms (e.g., microbial translocation and host immunity) underlying these differences.

## Author contributions

The authors’ responsibilities were as follows – GGH, BSA, SAli, ANM, CPS, FT, MA, SMN, MR, LU, BFA, PK, AEH, JMC, AJP, CNull, SPL, LCHF, AL: designed research; BSA, SAli, HOP, MZR, MA, GR, HND, JPB, CNekesa, CDA, SLF, MSH, PM, SAkther, JAG, STT, STA, TM, MKW, PC, AKS: conducted research; GGH, ANM, CH, STT: analyzed data or performed statistical analysis; GGH, BSA, SAli, ANM, CH, AL: wrote paper; GGH, BSA, SAli, ANM, AL: had primary responsibility for final content; and all authors: read and approved the final manuscript.

## Data availability

The corresponding author had full access to all study data as well as final responsibility around decision-making while submitting for publication.

## Funding

BSA, SAli, ANM, HOP, CPS, FT, MZR, MA, GR, HND, JPB, CNekesa, CDA, SLF, MSH, PM, SAkther, JAG, STA, TM, MKW, PC, SMN, AKS, MR, LU, BFA, PK, AEH, JMC, AJP, CNull, SPL, LCHF, and AL received funding for either salary or consulting fees through the Global Development grant (OPPGD759) from the Bill & Melinda Gates Foundation for this study. AL and STT received funding for salary through a grant from the National Institute of Allergy and Infectious Diseases of the NIH (grant number K01AI136885). The funder approved the design of the study. However, the funder played no role in data collection, analysis, interpretation or any decisions related to publication.

## Conflict of interest

AL reports financial support and travel were provided by Bill & Melinda Gates Foundation. AL reports financial support was provided by National Institute of Allergy and Infectious Diseases. BSA reports financial support was provided by Bill & Melinda Gates Foundation. SAli reports financial support was provided by Bill & Melinda Gates Foundation. ANM reports financial support and travel were provided by Bill & Melinda Gates Foundation. HOP reports financial support was provided by Bill & Melinda Gates Foundation. CPS reports financial support and travel were provided by Bill & Melinda Gates Foundation. FT reports financial support was provided by Bill & Melinda Gates Foundation. MZR reports financial support was provided by Bill & Melinda Gates Foundation. MA reports financial support was provided by Bill & Melinda Gates Foundation. GR reports financial support was provided by Bill & Melinda Gates Foundation. HND reports financial support and travel were provided by Bill & Melinda Gates Foundation. JPB reports financial support was provided by Bill & Melinda Gates Foundation. CNekesa reports financial support was provided by Bill & Melinda Gates Foundation. CDA reports financial support was provided by Bill & Melinda Gates Foundation. SLF reports financial support was provided by Bill & Melinda Gates Foundation. MSH reports financial support was provided by Bill & Melinda Gates Foundation. PM reports financial support was provided by Bill & Melinda Gates Foundation. SAkther reports financial support was provided by Bill & Melinda Gates Foundation. JAG reports financial support and travel were provided by Bill & Melinda Gates Foundation. STT reports financial support was provided by National Institute of Allergy and Infectious Diseases. STA reports financial support was provided by Bill & Melinda Gates Foundation. TM reports financial support was provided by Bill & Melinda Gates Foundation. MKW reports financial support was provided by Bill & Melinda Gates Foundation. PC reports financial support was provided by Bill & Melinda Gates Foundation. SMN reports financial support and travel were provided by Bill & Melinda Gates Foundation. AKS reports financial support was provided by Bill & Melinda Gates Foundation. MR reports financial support, equipment, drugs, or supplies, and travel were provided by Bill & Melinda Gates Foundation. LU reports financial support and travel were provided by Bill & Melinda Gates Foundation. BFA reports financial support was provided by Bill & Melinda Gates Foundation. PK reports financial support and travel were provided by Bill & Melinda Gates Foundation. AEH reports financial support was provided by Bill & Melinda Gates Foundation. JMC Jr. reports financial support, administrative support, equipment, drugs, or supplies, and travel were provided by Bill & Melinda Gates Foundation. AJP reports financial support and travel were provided by Bill & Melinda Gates Foundation. CNull reports financial support, administrative support, equipment, drugs, or supplies, and travel were provided by Bill & Melinda Gates Foundation. SPL reports financial support, administrative support, equipment, drugs, or supplies, and travel were provided by Bill & Melinda Gates Foundation. LCHF reports financial support and travel were provided by Bill & Melinda Gates Foundation. All other authors report no conflicts of interest.
