# Supplementary material for: Biomarkers of environmental enteric dysfunction and neurodevelopmental outcomes among children in rural Bangladesh and Kenya: a prospective cohort study
Source: Am J Clin Nutr. 2025 Jun 4;122(2):474–87. doi: 10.1016/j.ajcnut.2025.05.034 (PMC13168975; doi:10.1016/j.ajcnut.2025.05.034)

Environmental enteric dysfunction and neurodevelopmental outcomes among children in rural Bangladesh and Kenya: a prospective cohort study

Ho GG, Achando BS, Ali S, *et al.*

**Supplementary Materials**

**Table of Contents**

**Supplementary Figures**

Supplementary Figure 1: Flowchart of participants’ progress through enrollment, sample collection, and statistical analysis in Bangladesh. 2

Supplementary Figure 2: Flowchart of participants’ progress through enrollment, sample collection, and statistical analysis in Kenya. 3

**Supplementary Tables**

Supplementary Table 1: Covariates considered for inclusion in adjusted models 4

Supplementary Table 2: Enrollment characteristics within the WASH Benefits parent trial study population, EED and child development substudy population, and children lost to follow-up in Bangladesh 6

Supplementary Table 3: Enrollment characteristics within the WASH Benefits parent trial study population, EED and child development substudy population, and children lost to follow-up in Kenya 7

Supplementary Table 4: EED and Child Development in Bangladesh 8

Supplementary Table 5: EED and WHO Motor Milestones in Bangladesh (Hazard Ratios, Year 1) 12

Supplementary Table 6: Exclusive lactation sensitivity analysis in Bangladesh 14

Supplementary Table 7: EED and WHO Motor Milestones in Kenya (Hazard Ratios, Year 1) 17

Supplementary Table 8: EED and Child Development in Kenya 19

Supplementary Table 9: Exclusive lactation sensitivity analysis in Kenya 22

**Supplementary Appendices**

Supplementary Appendix A: Spline plots 24

Supplementary Appendix B: GLM-GAM comparison sensitivity analysis 55

Supplementary Appendix C: Diarrhea sensitivity analysis 61

**Supplementary Figures**


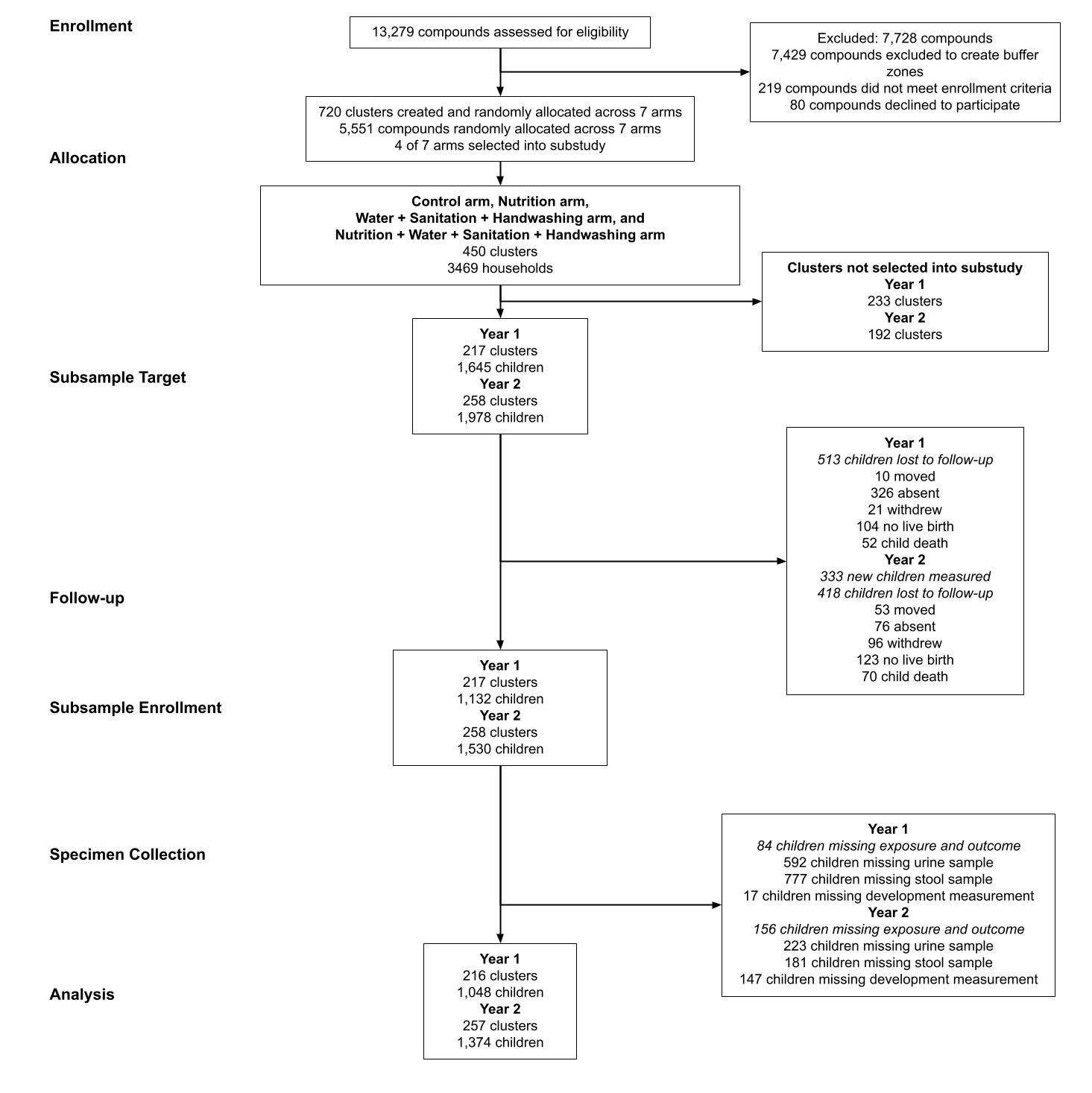
Supplementary Figure 1: **Flowchart of participants’ progress through enrollment, sample collection, and statistical analysis in Bangladesh**


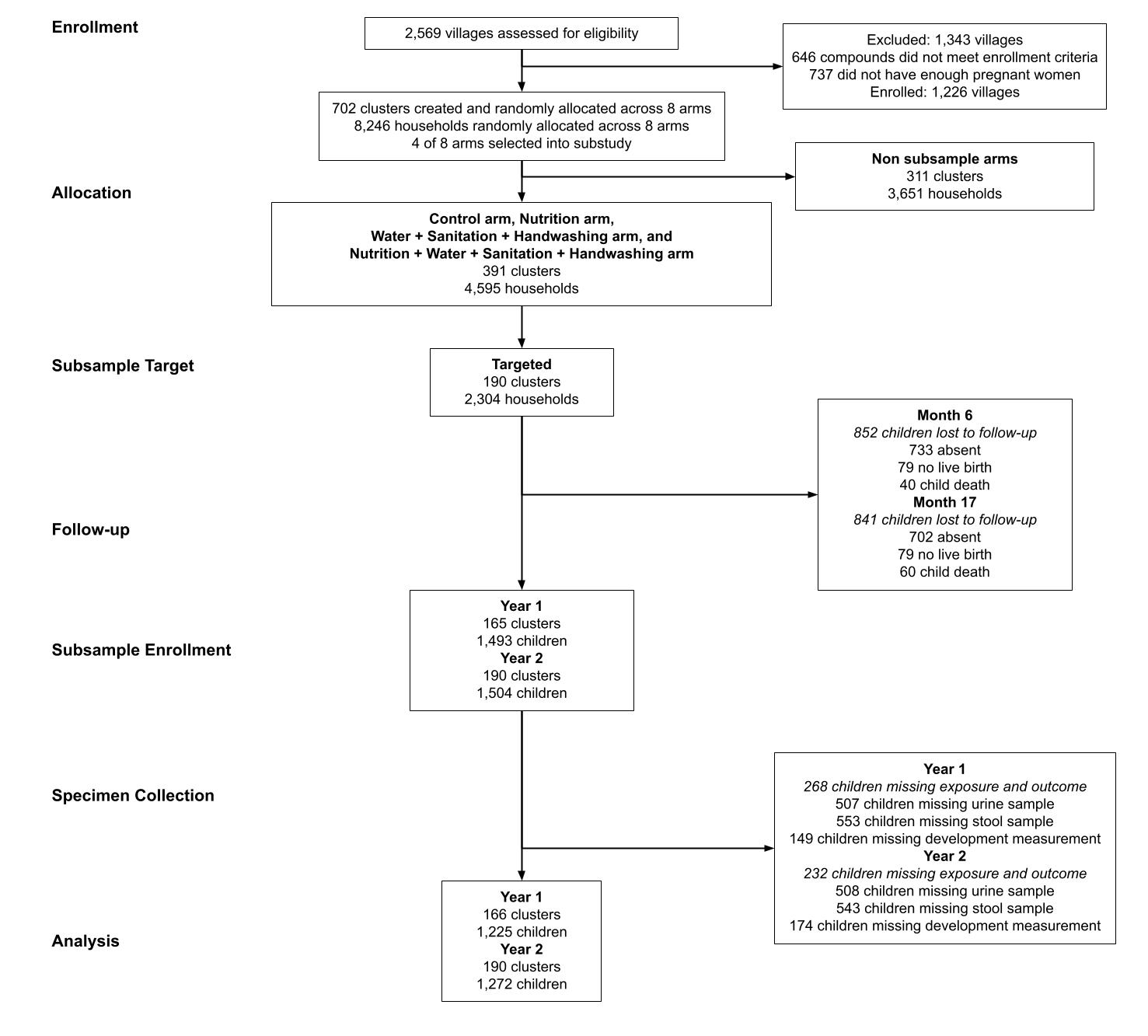
Supplementary Figure 2: **Flowchart of participants’ progress through enrollment, sample collection, and statistical analysis in Kenya**

Supplementary Table 1: **Covariates considered for inclusion in adjusted models**

| Covariate | Notes |
| --- | --- |
| Measured at Enrollment | |
| Child sex |  |
| Child birth order (first born, second born or greater) |  |
| Mother’s age (years) |  |
| Mother’s height (cm) |  |
| Mother’s education level (no education, primary, secondary) | Bangladesh: no education, primary, secondary  Kenya: none or incomplete primary, complete primary, any secondary |
| Household food insecurity (4-level Household Food Insecurity Access Scale categories) (Bangladesh only) |  |
| Household Hunger Scale (3-level Household Hunger Scale)  (Kenya only) |  |
| Number of children < 18 years in the household |  |
| Number of individuals living in the compound |  |
| Distance (in minutes) to the household’s primary drinking water source |  |
| Housing materials (each material tested separately) | Bangladesh: floor, walls, roof  Kenya: floor, roof |
| Asset-based household-wealth variable (continuous), calculated from the first principal component of a principal components analysis of the following household assets | Bangladesh: electricity, wardrobe, table, chair or bench, watch or clock, khat, chouki, working radio, working black/white or color television, refrigerator, bicycle (not child’s toy), motorcycle, sewing machine, mobile phone, land phone, number of cows, number of goats, number of chickens.  Kenya: electricity, clock, working radio, working black/white or color television, bicycle (not child’s toy), motorcycle, sewing machine, mobile phone, land phone, stove, number of cows, number of goats, number of dogs, number of poultry. |
| Measured at Follow-up | |
| Treatment arm (control; nutrition; combined water, sanitation, handwashing; nutrition plus combined water, sanitation, and handwashing). | Environmental enteric dysfunction biomarkers were collected in the active control arm in Kenya and the passive control arm in Bangladesh. |
| Child’s length-/height-for-age (LAZ/HAZ) and weight-for-age (WAZ) were tested as a potential confounder for subsequent time points |  |
| Month of measurement was tested as a potential confounder for concurrent and subsequent time points |  |
| Child age (in days) was tested as a potential confounder for concurrent and subsequent outcomes |  |
| The maternal Perceived Stress Scale (PSS) score at Follow-up 3 was assessed for confounding of Follow-up 3 outcomes in Kenya and Bangladesh. |  |
| The WHO Health and Life Experiences Survey, which measured maternal lifetime cumulative exposure to intimate partner violence was assessed for confounding of outcomes in Bangladesh only (this survey was not administered in Kenya). Intimate partner violence contributes to maternal depression and has been linked to elevated parenting stress and to harsher maternal parenting practices. |  |
| Maternal depressive symptoms | Bangladesh: Center for Epidemiologic Studies Depression (CES-D) Scale score 31 measured at Follow-up 2 was assessed for confounding of Follow-up 2 and 3 outcomes. The CES-D score measured at Follow-up 3 was assessed for confounding of Follow-up 3 outcomes. The CES-D score was a continuous measure, as no cutoff to identify depression has been validated in Bangladesh.  Kenya: Patient Health Questionnaire (PHQ) score measured at Follow-up 2 was assessed for confounding of Follow-up 2 and 3 outcomes, and the Follow-up 3 PHQ score was assessed for confounding of Follow-up 3 outcomes. |

Supplementary Table 2: **Enrollment characteristics within the WASH Benefits parent trial study population, EED and child development substudy population, and children lost to follow-up in Bangladesh**

|  | Full Study | Included | Lost to follow-up at Year 1 | Lost to follow-up at Year 2 |
| --- | --- | --- | --- | --- |
| Characteristic | N = 5,551^1,2^ | N = 1,592^1,2^ | N = 17^1,2^ | N = 129^1,2^ |
| Maternal Characteristics | | | | |
| Maternal age (years) | 23.00 (20.00, 27.00) | 24.00 (20.00, 27.00) | 24.00 (20.00, 25.00) | 22.00 (19.00, 25.00) |
| Maternal height (cm) | 150.60 (147.10, 154.10) | 150.38 (146.80, 154.15) | 149.60 (146.25, 151.15) | 150.98 (147.50, 153.98) |
| Maternal education completed |  |  |  |  |
| No education | 869 (15.65%) | 223 (14.01%) | 2 (11.76%) | 20 (15.50%) |
| Primary (1-5 years) | 1,742 (31.38%) | 451 (28.33%) | 6 (35.29%) | 36 (27.91%) |
| Secondary (>5 years) | 2,940 (52.96%) | 918 (57.66%) | 9 (52.94%) | 73 (56.59%) |
| Household Characteristics | | | | |
| Number of people per compound | 10 (6, 14) | 9 (6, 14) | 11 (8, 16) | 10 (6, 15) |
| Number of children <18 years in the household | 1 (1, 2) | 1 (1, 2) | 1 (1, 2) | 1 (1, 2) |
| Has electricity | 3,266 (58.84%) | 981 (61.62%) | 10 (58.82%) | 74 (57.36%) |
| Has a cement floor | 583 (10.50%) | 210 (13.19%) | 2 (11.76%) | 14 (10.85%) |
| Has an iron roof | 5,471 (98.56%) | 1,572 (98.74%) | 15 (88.24%) | 128 (99.22%) |
| Owns a mobile phone | 7 (0.13%) | 3 (0.19%) | 0 (0.00%) | 0 (0.00%) |
| Owns a motorcycle | 362 (6.52%) | 119 (7.47%) | 1 (5.88%) | 7 (5.43%) |
| Household food insecurity^3^ |  |  |  |  |
| Food Secure | 3,823 (68.87%) | 1,123 (70.54%) | 7 (41.18%) | 87 (67.44%) |
| Mildly Food Insecure | 492 (8.86%) | 132 (8.29%) | 1 (5.88%) | 10 (7.75%) |
| Moderately Food Insecure | 1,040 (18.74%) | 283 (17.78%) | 4 (23.53%) | 25 (19.38%) |
| Severely Food Insecure | 196 (3.53%) | 54 (3.39%) | 5 (29.41%) | 7 (5.43%) |
| Abbreviations: EED, environmental enteric dysfunction; Q1, first quartile; Q3, third quartile  ^1^Median (Q1, Q3); n (%) | | | | |
| ^2^N corresponds to households | | | | |
| ^3^Any level of food insecurity assessed using the Household Food Insecurity Access Scale | | | | |

Supplementary Table 3: **Enrollment characteristics within the WASH Benefits parent trial study population, EED and child development substudy population, and children lost to follow-up in Kenya**

|  | Full Study | Included | Lost to follow-up at Year 1 | Lost to follow-up at Year 2 |
| --- | --- | --- | --- | --- |
| Characteristic | N = 7,723^1,2^ | N = 1,703^1,2^ | N = 149^1,2^ | N = 174^1,2^ |
| Maternal Characteristics | | | | |
| Maternal age (years) | 25.01 (21.00, 29.91) | 25.52 (21.38, 30.01) | 24.38 (20.11, 28.86) | 23.99 (20.38, 29.14) |
| Maternal height (cm) | 160.10 (156.10, 164.30) | 160.50 (156.40, 164.40) | 161.00 (157.50, 165.00) | 159.60 (155.30, 162.80) |
| Maternal education completed |  |  |  |  |
| Primary | 1,747 (22.62%) | 403 (23.66%) | 32 (21.48%) | 42 (24.14%) |
| Incomplete Primary | 4,062 (52.60%) | 913 (53.61%) | 80 (53.69%) | 94 (54.02%) |
| Any Secondary | 1,905 (24.67%) | 385 (22.61%) | 37 (24.83%) | 38 (21.84%) |
| Missing | 9 (0.12%) | 2 (0.12%) | 0 (0.00%) | 0 (0.00%) |
| Household Characteristics | | | | |
| Number of people per compound | 7 (5, 10) | 7 (5, 11) | 7 (5, 12) | 7 (4, 10) |
| Number of children <18 years in the household | 3 (1, 4) | 3 (1, 4) | 3 (1, 4) | 2 (1, 4) |
| Has electricity | 529 (6.85%) | 102 (5.99%) | 6 (4.03%) | 8 (4.60%) |
| Has a cement floor | 432 (5.59%) | 85 (4.99%) | 9 (6.04%) | 6 (3.45%) |
| Has an iron roof | 5,101 (66.05%) | 1,050 (61.66%) | 100 (67.11%) | 103 (59.20%) |
| Owns a mobile phone | 6,133 (79.41%) | 1,398 (82.09%) | 121 (81.21%) | 130 (74.71%) |
| Owns a motorcycle | 688 (8.91%) | 149 (8.75%) | 17 (11.41%) | 16 (9.20%) |
| Prevalence of moderate to severe household hunger^3^ | 843 (10.92%) | 172 (10.10%) | 16 (10.74%) | 18 (10.34%) |
| Abbreviations: EED, environmental enteric dysfunction; Q1, first quartile; Q3, third quartile  ^1^Median (Q1, Q3); n (%) | | | | |
| ^2^N corresponds to households | | | | |
| ^3^Moderate to severe hunger defined using the Household Hunger Scale | | | | |

Supplementary Table 4: **EED and Child Development in Bangladesh**

| Outcome Subdomain | Exposure | n | Q1 | Q3 | Adj. Diff (95% CI)^1^ | Adj. P-value^1,2^ | FDR Corrected P-value^1,2^ |
| --- | --- | --- | --- | --- | --- | --- | --- |
| WHO Motor Milestones (Year 1) | | | | | | | |
| Sum Total | alpha-1 antitrypsin (3 mo) | 809 | -1.48 | -0.24 | 0.00 (-0.11, 0.12) | 0.94 | 0.98 |
| Milestones 2,4,5,6^3^ | alpha-1 antitrypsin (3 mo) | 809 | -1.48 | -0.24 | -0.03 (-0.23, 0.17) | 0.75 | 0.92 |
| Sum Total | myeloperoxidase (3 mo) | 811 | 8.35 | 9.85 | 0.06 (-0.07, 0.18) | 0.36 | 0.60 |
| Milestones 2,4,5,6^3^ | myeloperoxidase (3 mo) | 811 | 8.35 | 9.85 | 0.06 (-0.05, 0.16) | 0.28 | 0.51 |
| Sum Total | neopterin (3 mo) | 812 | 6.81 | 7.75 | 0.05 (-0.08, 0.18) | 0.44 | 0.60 |
| Milestones 2,4,5,6^3^ | neopterin (3 mo) | 812 | 6.81 | 7.75 | 0.07 (-0.05, 0.18) | 0.25 | 0.51 |
| Sum Total | lactulose (3 mo) | 988 | -1.55 | -0.01 | -0.25 (-0.50, -0.01) | 0.04** | 0.17* |
| Milestones 2,4,5,6^3^ | lactulose (3 mo) | 995 | -1.56 | -0.02 | -0.10 (-0.21, 0.00) | 0.05* | 0.17* |
| Sum Total | mannitol (3 mo) | 988 | 0.68 | 2.12 | -0.22 (-0.36, -0.08) | <0.001*** | 0.03** |
| Milestones 2,4,5,6^3^ | mannitol (3 mo) | 995 | 0.68 | 2.11 | -0.10 (-0.20, 0.01) | 0.06* | 0.17* |
| Communicative Development Inventory (Year 1) | | | | | | | |
| Receptive | alpha-1 antitrypsin (3 mo) | 800 | -1.48 | -0.23 | -0.03 (-0.25, 0.19) | 0.79 | 0.92 |
| Expressive without Language | alpha-1 antitrypsin (3 mo) | 796 | -1.48 | -0.22 | -0.07 (-0.24, 0.10) | 0.43 | 0.60 |
| Receptive | myeloperoxidase (3 mo) | 802 | 8.35 | 9.85 | -0.04 (-0.14, 0.06) | 0.45 | 0.60 |
| Expressive without Language | myeloperoxidase (3 mo) | 798 | 8.36 | 9.87 | 0.00 (-0.10, 0.10) | 0.98 | 0.98 |
| Receptive | neopterin (3 mo) | 803 | 6.80 | 7.75 | 0.23 (0.07, 0.39) | <0.001*** | 0.05** |
| Expressive without Language | neopterin (3 mo) | 799 | 6.80 | 7.75 | 0.03 (-0.19, 0.25) | 0.83 | 0.92 |
| Receptive | lactulose (3 mo) | 983 | -1.56 | -0.01 | -0.15 (-0.30, -0.01) | 0.04** | 0.17* |
| Expressive without Language | lactulose (3 mo) | 980 | -1.56 | -0.01 | -0.15 (-0.31, 0.01) | 0.06* | 0.17* |
| Receptive | mannitol (3 mo) | 983 | 0.67 | 2.11 | -0.12 (-0.26, 0.01) | 0.07* | 0.17* |
| Expressive without Language | mannitol (3 mo) | 980 | 0.67 | 2.12 | -0.12 (-0.28, 0.03) | 0.11* | 0.24 |
| Extended Ages and Stages (Year 2) | | | | | | | |
| Personal Social | alpha-1 antitrypsin (3 mo) | 698 | -1.45 | -0.21 | 0.02 (-0.08, 0.12) | 0.71 | 0.89 |
| Motor | alpha-1 antitrypsin (3 mo) | 689 | -1.44 | -0.21 | -0.04 (-0.13, 0.05) | 0.42 | 0.77 |
| Combined | alpha-1 antitrypsin (3 mo) | 696 | -1.45 | -0.21 | -0.10 (-0.27, 0.07) | 0.26 | 0.77 |
| Communication | alpha-1 antitrypsin (3 mo) | 696 | -1.45 | -0.21 | -0.08 (-0.24, 0.08) | 0.33 | 0.77 |
| Personal Social | myeloperoxidase (3 mo) | 699 | 8.38 | 9.91 | -0.06 (-0.18, 0.05) | 0.30 | 0.77 |
| Motor | myeloperoxidase (3 mo) | 690 | 8.41 | 9.91 | -0.03 (-0.14, 0.08) | 0.65 | 0.89 |
| Combined | myeloperoxidase (3 mo) | 697 | 8.39 | 9.91 | -0.06 (-0.17, 0.06) | 0.35 | 0.77 |
| Communication | myeloperoxidase (3 mo) | 697 | 8.39 | 9.91 | -0.05 (-0.08, -0.01) | 0.01** | 0.22 |
| Personal Social | neopterin (3 mo) | 700 | 6.83 | 7.76 | 0.07 (-0.04, 0.18) | 0.23 | 0.77 |
| Motor | neopterin (3 mo) | 691 | 6.83 | 7.75 | 0.07 (-0.05, 0.19) | 0.26 | 0.77 |
| Combined | neopterin (3 mo) | 698 | 6.83 | 7.77 | 0.10 (-0.04, 0.25) | 0.17* | 0.77 |
| Communication | neopterin (3 mo) | 698 | 6.83 | 7.76 | 0.19 (-0.01, 0.39) | 0.06* | 0.44 |
| Personal Social | lactulose (3 mo) | 868 | -1.57 | -0.03 | 0.02 (-0.21, 0.26) | 0.86 | 0.96 |
| Motor | lactulose (3 mo) | 861 | -1.56 | -0.02 | 0.05 (-0.05, 0.15) | 0.32 | 0.77 |
| Combined | lactulose (3 mo) | 866 | -1.57 | -0.03 | 0.03 (-0.07, 0.13) | 0.53 | 0.84 |
| Communication | lactulose (3 mo) | 867 | -1.57 | -0.03 | 0.03 (-0.06, 0.13) | 0.52 | 0.84 |
| Personal Social | mannitol (3 mo) | 868 | 0.69 | 2.11 | -0.02 (-0.12, 0.09) | 0.77 | 0.92 |
| Motor | mannitol (3 mo) | 861 | 0.69 | 2.12 | 0.04 (-0.06, 0.14) | 0.44 | 0.77 |
| Combined | mannitol (3 mo) | 866 | 0.69 | 2.11 | 0.03 (-0.08, 0.14) | 0.59 | 0.87 |
| Communication | mannitol (3 mo) | 867 | 0.69 | 2.11 | 0.02 (-0.08, 0.12) | 0.67 | 0.89 |
| Personal Social | alpha-1 antitrypsin (14 mo) | 1334 | -1.37 | -0.11 | 0.05 (-0.03, 0.12) | 0.22 | 0.77 |
| Motor | alpha-1 antitrypsin (14 mo) | 1315 | -1.37 | -0.11 | 0.01 (-0.17, 0.18) | 0.96 | 0.97 |
| Combined | alpha-1 antitrypsin (14 mo) | 1328 | -1.37 | -0.11 | 0.07 (-0.10, 0.24) | 0.41 | 0.77 |
| Communication | alpha-1 antitrypsin (14 mo) | 1334 | -1.37 | -0.11 | 0.04 (-0.11, 0.19) | 0.58 | 0.87 |
| Personal Social | myeloperoxidase (14 mo) | 1334 | 7.91 | 9.24 | -0.01 (-0.09, 0.06) | 0.71 | 0.89 |
| Motor | myeloperoxidase (14 mo) | 1315 | 7.91 | 9.24 | -0.02 (-0.09, 0.05) | 0.62 | 0.88 |
| Combined | myeloperoxidase (14 mo) | 1328 | 7.91 | 9.24 | 0.00 (-0.07, 0.06) | 0.90 | 0.96 |
| Communication | myeloperoxidase (14 mo) | 1334 | 7.91 | 9.24 | 0.00 (-0.06, 0.07) | 0.91 | 0.96 |
| Personal Social | neopterin (14 mo) | 1334 | 6.48 | 7.64 | 0.00 (-0.11, 0.10) | 0.96 | 0.97 |
| Motor | neopterin (14 mo) | 1315 | 6.47 | 7.64 | 0.03 (-0.04, 0.10) | 0.38 | 0.77 |
| Combined | neopterin (14 mo) | 1328 | 6.48 | 7.64 | 0.05 (-0.04, 0.14) | 0.30 | 0.77 |
| Communication | neopterin (14 mo) | 1334 | 6.48 | 7.64 | 0.05 (-0.04, 0.14) | 0.24 | 0.77 |
| Personal Social | lactulose (14 mo) | 1299 | -1.91 | -0.52 | -0.06 (-0.18, 0.07) | 0.40 | 0.77 |
| Motor | lactulose (14 mo) | 1284 | -1.90 | -0.52 | -0.14 (-0.26, -0.01) | 0.03** | 0.30 |
| Combined | lactulose (14 mo) | 1294 | -1.91 | -0.52 | -0.14 (-0.28, 0.00) | 0.04** | 0.38 |
| Communication | lactulose (14 mo) | 1299 | -1.91 | -0.52 | -0.07 (-0.19, 0.05) | 0.26 | 0.77 |
| Personal Social | mannitol (14 mo) | 1299 | 0.03 | 1.54 | 0.01 (-0.06, 0.08) | 0.78 | 0.92 |
| Motor | mannitol (14 mo) | 1284 | 0.03 | 1.54 | -0.22 (-0.36, -0.07) | <0.001*** | 0.10* |
| Combined | mannitol (14 mo) | 1294 | 0.03 | 1.54 | -0.05 (-0.18, 0.08) | 0.48 | 0.81 |
| Communication | mannitol (14 mo) | 1299 | 0.03 | 1.54 | 0.03 (-0.03, 0.10) | 0.34 | 0.77 |
| Personal Social | regenerating gene 1β (14 mo) | 1332 | 4.70 | 5.48 | -0.01 (-0.05, 0.03) | 0.69 | 0.86 |
| Motor | regenerating gene 1β (14 mo) | 1313 | 4.70 | 5.48 | 0.02 (-0.02, 0.06) | 0.35 | 0.70 |
| Combined | regenerating gene 1β (14 mo) | 1326 | 4.71 | 5.48 | 0.01 (-0.03, 0.05) | 0.72 | 0.86 |
| Communication | regenerating gene 1β (14 mo) | 1332 | 4.71 | 5.48 | 0.00 (-0.04, 0.04) | 0.97 | 0.97 |
| Communicative Development Inventory (Year 2) | | | | | | | |
| Receptive | alpha-1 antitrypsin (3 mo) | 703 | -1.45 | -0.21 | -0.02 (-0.11, 0.07) | 0.65 | 0.89 |
| Expressive without Language | alpha-1 antitrypsin (3 mo) | 709 | -1.45 | -0.21 | 0.00 (0.00, 0.00) | <0.001*** | 0.10* |
| Receptive | myeloperoxidase (3 mo) | 704 | 8.38 | 9.91 | -0.01 (-0.12, 0.09) | 0.79 | 0.92 |
| Expressive without Language | myeloperoxidase (3 mo) | 710 | 8.37 | 9.90 | 0.00 (-0.11, 0.11) | 1.00 | 1.00 |
| Receptive | neopterin (3 mo) | 705 | 6.83 | 7.75 | 0.08 (-0.02, 0.18) | 0.10* | 0.55 |
| Expressive without Language | neopterin (3 mo) | 711 | 6.82 | 7.75 | 0.09 (-0.02, 0.20) | 0.10* | 0.55 |
| Receptive | lactulose (3 mo) | 873 | -1.56 | -0.03 | 0.06 (-0.15, 0.27) | 0.57 | 0.87 |
| Expressive without Language | lactulose (3 mo) | 881 | -1.57 | -0.03 | 0.09 (-0.13, 0.31) | 0.43 | 0.77 |
| Receptive | mannitol (3 mo) | 873 | 0.69 | 2.10 | 0.01 (-0.17, 0.20) | 0.91 | 0.96 |
| Expressive without Language | mannitol (3 mo) | 881 | 0.69 | 2.10 | -0.02 (-0.22, 0.18) | 0.86 | 0.96 |
| Receptive | alpha-1 antitrypsin (14 mo) | 1337 | -1.36 | -0.11 | 0.19 (0.03, 0.35) | 0.02** | 0.30 |
| Expressive without Language | alpha-1 antitrypsin (14 mo) | 1354 | -1.36 | -0.11 | 0.19 (0.02, 0.36) | 0.02** | 0.30 |
| Receptive | myeloperoxidase (14 mo) | 1337 | 7.91 | 9.25 | -0.01 (-0.08, 0.05) | 0.70 | 0.89 |
| Expressive without Language | myeloperoxidase (14 mo) | 1354 | 7.91 | 9.24 | -0.06 (-0.13, 0.00) | 0.07* | 0.44 |
| Receptive | neopterin (14 mo) | 1337 | 6.48 | 7.64 | 0.04 (-0.02, 0.10) | 0.23 | 0.77 |
| Expressive without Language | neopterin (14 mo) | 1354 | 6.48 | 7.64 | 0.05 (-0.01, 0.12) | 0.11* | 0.57 |
| Receptive | lactulose (14 mo) | 1301 | -1.91 | -0.51 | -0.04 (-0.12, 0.04) | 0.32 | 0.77 |
| Expressive without Language | lactulose (14 mo) | 1317 | -1.91 | -0.52 | -0.04 (-0.12, 0.05) | 0.41 | 0.77 |
| Receptive | mannitol (14 mo) | 1301 | 0.03 | 1.54 | 0.03 (-0.05, 0.12) | 0.48 | 0.81 |
| Expressive without Language | mannitol (14 mo) | 1317 | 0.03 | 1.54 | 0.00 (-0.07, 0.08) | 0.91 | 0.96 |
| Receptive | regenerating gene 1β (14 mo) | 1335 | 4.71 | 5.48 | -0.03 (-0.07, 0.01) | 0.13* | 0.40 |
| Expressive without Language | regenerating gene 1β (14 mo) | 1352 | 4.71 | 5.48 | -0.04 (-0.08, 0.00) | 0.07* | 0.40 |
| Abbreviations: EED, environmental enteric dysfunction; mo, months; Q1, first quartile; Q3, third quartile; Adj Diff, adjusted difference in outcome between the 25^th^ and 75^th^ percentile of exposure; Adj P-value, P-value of adjusted difference between the 25^th^ and 75^th^ percentile of exposure; FDR, false discovery rate  ^1^Adjusted for prespecified and prescreened covariates: child sex, child age, child birth order, mother’s age, mother’s height, mother’s education, household food security, number of children < 18 years old in the household, number of people living in the compound, distance (in minutes) to the primary water source, household materials (floor, roof), asset-based household wealth (electricity, clock, working radio, working black/white or color television, bicycle, motorcycle, sewing machine, mobile phone, land phone, stove, number of cows, number of goats, number of dogs, and number of poultry), treatment arm, prior length-for-age Z-score and weight-for-age Z-score, month of measurement, mother’s perceived stress (only for year 2 outcomes), mother’s depressive symptoms, and mother’s exposure to intimate partner violence (IPV) during lifetime. See Supplementary Table 1 for more details. | | | | | | | |
| ^2^Asterisk denotes level of significance: *** <0.001, ** <0.05, * <0.2 | | | | | | | |
| ^3^Milestones 2,4,5,6 correspond to hands-and-knees crawling, walking with assistance, standing alone, and walking alone, respectively. | | | | | | | |

Supplementary Table 5: **EED and WHO Motor Milestones in Bangladesh (Hazard Ratios, Year 1)**

| WHO Motor Milestones | Exposure | n | Q1 | Q3 | Adj. Diff (95% CI)^1,2^ | Adj. P-value^1,3^ | FDR Corrected P-value^1,3^ | |
| --- | --- | --- | --- | --- | --- | --- | --- | --- |
| Sitting without support | alpha-1 antitrypsin (3 mo) | 803 | -1.47 | -0.24 | --^ | 0.90 | 0.95 | |
| Hands-and-knees crawling | alpha-1 antitrypsin (3 mo) | 800 | -1.47 | -0.24 | 0.97 (0.87, 1.09) | 0.68 | 0.85 | |
| Standing with assistance | alpha-1 antitrypsin (3 mo) | 802 | -1.48 | -0.24 | 0.82 (0.66, 1.02) | 0.07* | 0.36 | |
| Walking with assistance | alpha-1 antitrypsin (3 mo) | 801 | -1.48 | -0.25 | 1.06 (0.94, 1.21) | 0.36 | 0.67 | |
| Standing alone | alpha-1 antitrypsin (3 mo) | 803 | -1.48 | -0.24 | 0.99 (0.72, 1.38) | 0.98 | 0.98 | |
| Walking alone | alpha-1 antitrypsin (3 mo) | 797 | -1.47 | -0.24 | 1.03 (0.66, 1.60) | 0.92 | 0.95 | |
| Sitting without support | myeloperoxidase (3 mo) | 805 | 8.38 | 9.87 | --^ | 0.60 | 0.83 | |
| Hands-and-knees crawling | myeloperoxidase (3 mo) | 802 | 8.38 | 9.88 | 0.93 (0.79, 1.09) | 0.40 | 0.71 | |
| Standing with assistance | myeloperoxidase (3 mo) | 804 | 8.37 | 9.87 | 0.96 (0.82, 1.12) | 0.61 | 0.83 | |
| Walking with assistance | myeloperoxidase (3 mo) | 803 | 8.36 | 9.86 | 1.04 (0.90, 1.20) | 0.59 | 0.83 | |
| Standing alone | myeloperoxidase (3 mo) | 805 | 8.37 | 9.87 | 1.03 (0.87, 1.21) | 0.77 | 0.92 | |
| Walking alone | myeloperoxidase (3 mo) | 799 | 8.40 | 9.88 | 1.12 (0.91, 1.38) | 0.28 | 0.56 | |
| Sitting without support | neopterin (3 mo) | 806 | 6.81 | 7.75 | --^ | 0.59 | 0.83 | |
| Hands-and-knees crawling | neopterin (3 mo) | 803 | 6.81 | 7.75 | 0.92 (0.81, 1.04) | 0.19* | 0.50 | |
| Standing with assistance | neopterin (3 mo) | 805 | 6.81 | 7.75 | 0.94 (0.79, 1.12) | 0.53 | 0.83 | |
| Walking with assistance | neopterin (3 mo) | 804 | 6.81 | 7.75 | 1.25 (1.00, 1.56) | 0.04** | 0.30 | |
| Standing alone | neopterin (3 mo) | 806 | 6.81 | 7.75 | 1.10 (0.93, 1.30) | 0.25 | 0.56 | |
| Walking alone | neopterin (3 mo) | 800 | 6.81 | 7.75 | 0.96 (0.64, 1.45) | 0.86 | 0.95 | |
| Sitting without support | lactulose (3 mo) | 987 | -1.55 | -0.01 | --^ | 0.82 | 0.95 | |
| Hands-and-knees crawling | lactulose (3 mo) | 984 | -1.55 | 0.00 | 0.82 (0.65, 1.03) | 0.08* | 0.36 | |
| Standing with assistance | lactulose (3 mo) | 987 | -1.56 | -0.01 | 0.90 (0.78, 1.04) | 0.15* | 0.44 | |
| Walking with assistance | lactulose (3 mo) | 986 | -1.56 | -0.01 | 0.83 (0.62, 1.10) | 0.20* | 0.50 | |
| Standing alone | lactulose (3 mo) | 980 | -1.55 | 0.00 | 0.67 (0.47, 0.96) | 0.03** | 0.30 | |
| Walking alone | lactulose (3 mo) | 980 | -1.55 | 0.00 | 0.89 (0.72, 1.09) | 0.27 | 0.56 | |
| Sitting without support | mannitol (3 mo) | 987 | 0.68 | 2.12 | 0.59 (0.30, 1.16) | 0.12* | 0.42 | |
| Hands-and-knees crawling | mannitol (3 mo) | 984 | 0.68 | 2.12 | 0.74 (0.64, 0.86) | <0.001*** | <0.001*** | |
| Standing with assistance | mannitol (3 mo) | 987 | 0.68 | 2.11 | 0.85 (0.72, 1.00) | 0.05* | 0.30 | |
| Walking with assistance | mannitol (3 mo) | 986 | 0.67 | 2.11 | 0.90 (0.78, 1.03) | 0.13* | 0.42 | |
| Standing alone | mannitol (3 mo) | 980 | 0.67 | 2.12 | 0.79 (0.63, 0.99) | 0.04** | 0.30 | |
| Walking alone | mannitol (3 mo) | 980 | 0.67 | 2.12 | 0.96 (0.79, 1.16) | 0.67 | 0.85 | |
| Abbreviations: EED, environmental enteric dysfunction; mo, months; Q1, first quartile; Q3, third quartile; Adj Diff, adjusted difference in outcome between the 25^th^ and 75^th^ percentile of exposure; Adj P-value, P-value of adjusted difference between the 25^th^ and 75^th^ percentile of exposure; FDR, false discovery rate  ^1^Adjusted for prespecified and prescreened covariates: child sex, child age, child birth order, mother’s age, mother’s height, mother’s education, household food security, number of children < 18 years old in the household, number of people living in the compound, distance (in minutes) to the primary water source, household materials (floor, roof), asset-based household wealth (electricity, clock, working radio, working black/white or color television, bicycle, motorcycle, sewing machine, mobile phone, land phone, stove, number of cows, number of goats, number of dogs, and number of poultry), treatment arm, prior length-for-age Z-score and weight-for-age Z-score, month of measurement, mother’s depressive symptoms, and mother’s exposure to intimate partner violence (IPV) during lifetime. See Supplementary Table 1 for more details. | | | | | | | |  |
| ^2^--^ indicates hazard ratio not calculated due to data sparsity. | | | | | | | |  |
| ^3^Asterisk denotes level of significance: *** <0.001, ** <0.05, * <0.2 | | | | | | | |  |

Supplementary Table 6: **Exclusive Lactation Sensitivity Analysis in Bangladesh**

|  |  | Exclusive Lactation Variable Included | | | Exclusive Lactation Variable Not Included | | |
| --- | --- | --- | --- | --- | --- | --- | --- |
| Outcome Subdomain | Exposure | n^1^ | Adj. Diff (95% CI)^1^ | Adj. P-value^1,2^ | n^1^ | Adj. Diff (95% CI)^1^ | Adj. P-value^1,2^ |
| WHO Motor Milestones (Year 1) | | | | | | | |
| Sum Total | alpha-1 antitrypsin (3 mo) | 745 | -0.03 (-0.25, 0.19) | 0.77 | 809 | 0.00 (-0.11, 0.12) | 0.94 |
| Milestones 2,4,5,6^3^ | alpha-1 antitrypsin (3 mo) | 745 | 0.01 (-0.08, 0.11) | 0.80 | 809 | -0.03 (-0.23, 0.17) | 0.75 |
| Sum Total | myeloperoxidase (3 mo) | 747 | 0.06 (-0.07, 0.19) | 0.36 | 811 | 0.06 (-0.07, 0.18) | 0.36 |
| Milestones 2,4,5,6^3^ | myeloperoxidase (3 mo) | 747 | 0.07 (-0.04, 0.18) | 0.22 | 811 | 0.06 (-0.05, 0.16) | 0.28 |
| Sum Total | neopterin (3 mo) | 748 | 0.06 (-0.08, 0.21) | 0.39 | 812 | 0.05 (-0.08, 0.18) | 0.44 |
| Milestones 2,4,5,6^3^ | neopterin (3 mo) | 748 | 0.18 (-0.04, 0.39) | 0.11* | 812 | 0.07 (-0.05, 0.18) | 0.25 |
| Sum Total | lactulose (3 mo) | 917 | -0.22 (-0.47, 0.03) | 0.09* | 988 | -0.25 (-0.50, -0.01) | 0.04** |
| Milestones 2,4,5,6^3^ | lactulose (3 mo) | 923 | -0.10 (-0.23, 0.03) | 0.14* | 995 | -0.10 (-0.21, 0.00) | 0.05* |
| Sum Total | mannitol (3 mo) | 917 | -0.23 (-0.38, -0.07) | <0.01*** | 988 | -0.22 (-0.36, -0.08) | <0.01*** |
| Milestones 2,4,5,6^3^ | mannitol (3 mo) | 923 | -0.10 (-0.22, 0.02) | 0.12* | 995 | -0.10 (-0.20, 0.01) | 0.06* |
| Communicative Development Inventory (Year 1) | | | | | | | |
| Receptive | alpha-1 antitrypsin (3 mo) | 736 | -0.04 (-0.27, 0.20) | 0.77 | 800 | -0.03 (-0.25, 0.19) | 0.79 |
| Expressive without Language | alpha-1 antitrypsin (3 mo) | 733 | -0.07 (-0.24, 0.11) | 0.45 | 796 | -0.07 (-0.24, 0.10) | 0.43 |
| Receptive | myeloperoxidase (3 mo) | 738 | -0.03 (-0.14, 0.08) | 0.59 | 802 | -0.04 (-0.14, 0.06) | 0.45 |
| Expressive without Language | myeloperoxidase (3 mo) | 735 | -0.01 (-0.09, 0.07) | 0.81 | 798 | 0.00 (-0.10, 0.10) | 0.98 |
| Receptive | neopterin (3 mo) | 739 | 0.19 (0.04, 0.33) | 0.01** | 803 | 0.23 (0.07, 0.39) | <0.01*** |
| Expressive without Language | neopterin (3 mo) | 736 | 0.07 (-0.16, 0.29) | 0.57 | 799 | 0.03 (-0.19, 0.25) | 0.83 |
| Receptive | lactulose (3 mo) | 911 | -0.13 (-0.26, 0.00) | 0.06* | 983 | -0.15 (-0.30, -0.01) | 0.04** |
| Expressive without Language | lactulose (3 mo) | 908 | -0.11 (-0.26, 0.04) | 0.14* | 980 | -0.15 (-0.31, 0.01) | 0.06* |
| Receptive | mannitol (3 mo) | 911 | -0.13 (-0.26, 0.01) | 0.06* | 983 | -0.12 (-0.26, 0.01) | 0.07* |
| Expressive without Language | mannitol (3 mo) | 908 | -0.11 (-0.26, 0.05) | 0.17* | 980 | -0.12 (-0.28, 0.03) | 0.11* |
| Extended Ages and Stages (Year 2) | | | | | | | |
| Personal Social | alpha-1 antitrypsin (3 mo) | 627 | 0.01 (-0.18, 0.20) | 0.93 | 698 | 0.02 (-0.08, 0.12) | 0.71 |
| Motor | alpha-1 antitrypsin (3 mo) | 619 | -0.04 (-0.13, 0.06) | 0.44 | 689 | -0.04 (-0.13, 0.05) | 0.42 |
| Combined | alpha-1 antitrypsin (3 mo) | 625 | -0.11 (-0.29, 0.07) | 0.24 | 696 | -0.10 (-0.27, 0.07) | 0.26 |
| Communication | alpha-1 antitrypsin (3 mo) | 625 | -0.06 (-0.21, 0.09) | 0.43 | 696 | -0.08 (-0.24, 0.08) | 0.33 |
| Personal Social | myeloperoxidase (3 mo) | 628 | -0.09 (-0.21, 0.03) | 0.12* | 699 | -0.06 (-0.18, 0.05) | 0.30 |
| Motor | myeloperoxidase (3 mo) | 620 | -0.07 (-0.22, 0.08) | 0.35 | 690 | -0.03 (-0.14, 0.08) | 0.65 |
| Combined | myeloperoxidase (3 mo) | 626 | -0.08 (-0.20, 0.04) | 0.19* | 697 | -0.06 (-0.17, 0.06) | 0.35 |
| Communication | myeloperoxidase (3 mo) | 626 | -0.04 (-0.08, 0.00) | 0.04** | 697 | -0.05 (-0.08, -0.01) | 0.01** |
| Personal Social | neopterin (3 mo) | 629 | 0.11 (0.00, 0.22) | 0.06* | 700 | 0.07 (-0.04, 0.18) | 0.23 |
| Motor | neopterin (3 mo) | 621 | 0.14 (-0.07, 0.36) | 0.19* | 691 | 0.07 (-0.05, 0.19) | 0.26 |
| Combined | neopterin (3 mo) | 627 | 0.13 (-0.04, 0.29) | 0.14* | 698 | 0.10 (-0.04, 0.25) | 0.17* |
| Communication | neopterin (3 mo) | 627 | 0.06 (-0.11, 0.22) | 0.51 | 698 | 0.19 (-0.01, 0.39) | 0.06* |
| Personal Social | lactulose (3 mo) | 791 | -0.14 (-0.39, 0.11) | 0.27 | 868 | 0.02 (-0.21, 0.26) | 0.86 |
| Motor | lactulose (3 mo) | 785 | 0.00 (-0.09, 0.10) | 0.98 | 861 | 0.05 (-0.05, 0.15) | 0.32 |
| Combined | lactulose (3 mo) | 789 | 0.00 (-0.10, 0.10) | 0.97 | 866 | 0.03 (-0.07, 0.13) | 0.53 |
| Communication | lactulose (3 mo) | 790 | 0.01 (-0.09, 0.10) | 0.87 | 867 | 0.03 (-0.06, 0.13) | 0.52 |
| Personal Social | mannitol (3 mo) | 791 | -0.06 (-0.20, 0.08) | 0.41 | 868 | -0.02 (-0.12, 0.09) | 0.77 |
| Motor | mannitol (3 mo) | 785 | 0.01 (-0.09, 0.10) | 0.92 | 861 | 0.04 (-0.06, 0.14) | 0.44 |
| Combined | mannitol (3 mo) | 789 | 0.01 (-0.09, 0.12) | 0.84 | 866 | 0.03 (-0.08, 0.14) | 0.59 |
| Communication | mannitol (3 mo) | 790 | 0.01 (-0.09, 0.11) | 0.85 | 867 | 0.02 (-0.08, 0.12) | 0.67 |
| Communicative Development Inventory (Year 2) | | | | | | | |
| Receptive | alpha-1 antitrypsin (3 mo) | 632 | -0.03 (-0.12, 0.06) | 0.54 | 703 | -0.02 (-0.11, 0.07) | 0.65 |
| Expressive without Language | alpha-1 antitrypsin (3 mo) | 637 | 0.00 (0.00, 0.00) | 0.05* | 709 | 0.00 (0.00, 0.00) | <0.01*** |
| Receptive | myeloperoxidase (3 mo) | 633 | 0.01 (-0.10, 0.11) | 0.88 | 704 | -0.01 (-0.12, 0.09) | 0.79 |
| Expressive without Language | myeloperoxidase (3 mo) | 638 | 0.00 (0.00, 0.00) | 0.04** | 710 | 0.00 (-0.11, 0.11) | 1.00 |
| Receptive | neopterin (3 mo) | 634 | 0.07 (-0.03, 0.17) | 0.17* | 705 | 0.08 (-0.02, 0.18) | 0.10* |
| Expressive without Language | neopterin (3 mo) | 639 | 0.05 (0.01, 0.10) | 0.03** | 711 | 0.09 (-0.02, 0.20) | 0.10* |
| Receptive | lactulose (3 mo) | 796 | 0.04 (-0.16, 0.25) | 0.71 | 873 | 0.06 (-0.15, 0.27) | 0.57 |
| Expressive without Language | lactulose (3 mo) | 803 | 0.00 (0.00, 0.00) | <0.01*** | 881 | 0.09 (-0.13, 0.31) | 0.43 |
| Receptive | mannitol (3 mo) | 796 | 0.04 (-0.06, 0.13) | 0.47 | 873 | 0.01 (-0.17, 0.20) | 0.91 |
| Expressive without Language | mannitol (3 mo) | 803 | -0.08 (-0.27, 0.12) | 0.44 | 881 | -0.02 (-0.22, 0.18) | 0.86 |

| Abbreviations: EED, environmental enteric dysfunction; mo, months; Adj Diff, adjusted difference in outcome between the 25^th^ and 75^th^ percentile of exposure; Adj P-value, P-value of adjusted difference between the 25^th^ and 75^th^ percentile of exposure  ^1^Adjusted for prespecified and prescreened covariates: child sex, child age, child birth order, mother’s age, mother’s height, mother’s education, household food security, number of children < 18 years old in the household, number of people living in the compound, distance (in minutes) to the primary water source, household materials (floor, roof), asset-based household wealth (electricity, clock, working radio, working black/white or color television, bicycle, motorcycle, sewing machine, mobile phone, land phone, stove, number of cows, number of goats, number of dogs, and number of poultry), treatment arm, prior length-for-age Z-score and weight-for-age Z-score, month of measurement, mother’s perceived stress (only for year 2 outcomes), mother’s depressive symptoms, and mother’s exposure to intimate partner violence (IPV) during lifetime. See Supplementary Table 1 for more details. |
| --- |
| ^2^Asterisk denotes level of significance: *** <0.01, ** <0.05, * <0.2 |
| ^3^Milestones 2,4,5,6 correspond to hands-and-knees crawling, walking with assistance, standing alone, and walking alone, respectively. |

Supplementary Table 7: **EED and WHO Motor Milestones in Kenya (Hazard Ratios, Year 1)**

| WHO Motor Milestones | Exposure | n | Q1 | Q3 | Adj. Diff (95% CI)^1,2^ | Adj. P-value^1,3^ | FDR Corrected P-value^1,3^ |
| --- | --- | --- | --- | --- | --- | --- | --- |
| Sitting without support | alpha-1 antitrypsin (6 mo) | 1001 | -1.30 | -0.03 | --^ | 0.94 | 0.96 |
| Hands-and-knees crawling | alpha-1 antitrypsin (6 mo) | 973 | -1.30 | -0.03 | 0.36 (0.17, 0.76) | 0.01*** | 0.23 |
| Standing with assistance | alpha-1 antitrypsin (6 mo) | 1005 | -1.30 | -0.03 | 1.03 (0.90, 1.17) | 0.72 | 0.96 |
| Walking with assistance | alpha-1 antitrypsin (6 mo) | 979 | -1.29 | -0.03 | 0.96 (0.85, 1.10) | 0.58 | 0.96 |
| Standing alone | alpha-1 antitrypsin (6 mo) | 997 | -1.31 | -0.03 | 1.08 (0.95, 1.24) | 0.23 | 0.68 |
| Walking alone | alpha-1 antitrypsin (6 mo) | 978 | -1.30 | -0.03 | 1.10 (0.95, 1.27) | 0.22 | 0.68 |
| Sitting without support | myeloperoxidase (6 mo) | 1002 | 7.41 | 8.90 | 0.90 (0.53, 1.51) | 0.70 | 0.96 |
| Hands-and-knees crawling | myeloperoxidase (6 mo) | 974 | 7.41 | 8.90 | 0.89 (0.74, 1.06) | 0.19* | 0.68 |
| Standing with assistance | myeloperoxidase (6 mo) | 1006 | 7.41 | 8.90 | 1.04 (0.92, 1.18) | 0.54 | 0.96 |
| Walking with assistance | myeloperoxidase (6 mo) | 980 | 7.41 | 8.90 | 1.04 (0.89, 1.20) | 0.64 | 0.96 |
| Standing alone | myeloperoxidase (6 mo) | 998 | 7.41 | 8.90 | 1.02 (0.90, 1.15) | 0.82 | 0.96 |
| Walking alone | myeloperoxidase (6 mo) | 979 | 7.41 | 8.90 | 0.97 (0.85, 1.11) | 0.66 | 0.96 |
| Sitting without support | neopterin (6 mo) | 1003 | 7.10 | 7.96 | 0.58 (0.29, 1.16) | 0.12* | 0.68 |
| Hands-and-knees crawling | neopterin (6 mo) | 975 | 7.11 | 7.96 | 1.01 (0.86, 1.18) | 0.94 | 0.96 |
| Standing with assistance | neopterin (6 mo) | 1007 | 7.10 | 7.96 | 1.08 (0.97, 1.21) | 0.15* | 0.68 |
| Walking with assistance | neopterin (6 mo) | 981 | 7.11 | 7.96 | 1.01 (0.90, 1.13) | 0.90 | 0.96 |
| Standing alone | neopterin (6 mo) | 999 | 7.11 | 7.96 | 1.08 (0.88, 1.33) | 0.48 | 0.96 |
| Walking alone | neopterin (6 mo) | 980 | 7.10 | 7.96 | 1.51 (1.06, 2.13) | 0.02** | 0.31 |
| Sitting without support | lactulose (6 mo) | 1083 | -2.24 | -0.85 | --^ | 0.91 | 0.96 |
| Hands-and-knees crawling | lactulose (6 mo) | 1076 | -2.25 | -0.85 | 0.90 (0.76, 1.06) | 0.22 | 0.68 |
| Standing with assistance | lactulose (6 mo) | 1083 | -2.25 | -0.85 | 0.95 (0.84, 1.06) | 0.34 | 0.93 |
| Walking with assistance | lactulose (6 mo) | 1081 | -2.25 | -0.85 | 1.00 (0.89, 1.12) | 0.96 | 0.96 |
| Standing alone | lactulose (6 mo) | 1078 | -2.24 | -0.85 | 1.08 (0.96, 1.22) | 0.22 | 0.68 |
| Walking alone | lactulose (6 mo) | 1083 | -2.24 | -0.85 | 1.02 (0.89, 1.16) | 0.82 | 0.96 |
| Sitting without support | mannitol (6 mo) | 1083 | -0.06 | 1.29 | --^ | 0.83 | 0.96 |
| Hands-and-knees crawling | mannitol (6 mo) | 1076 | -0.06 | 1.29 | 0.99 (0.85, 1.15) | 0.88 | 0.96 |
| Standing with assistance | mannitol (6 mo) | 1083 | -0.06 | 1.29 | 0.97 (0.86, 1.08) | 0.57 | 0.96 |
| Walking with assistance | mannitol (6 mo) | 1081 | -0.06 | 1.29 | 0.99 (0.88, 1.11) | 0.87 | 0.96 |
| Standing alone | mannitol (6 mo) | 1078 | -0.06 | 1.30 | 1.09 (0.97, 1.23) | 0.15* | 0.68 |
| Walking alone | mannitol (6 mo) | 1083 | -0.06 | 1.29 | 1.04 (0.91, 1.19) | 0.55 | 0.96 |
| Abbreviations: EED, environmental enteric dysfunction; mo, months; Q1, first quartile; Q3, third quartile; Adj Diff, adjusted difference in outcome between the 25^th^ and 75^th^ percentile of exposure; Adj P-value, P-value of adjusted difference between the 25^th^ and 75^th^ percentile of exposure; FDR, false discovery rate  ^1^Adjusted for prespecified and prescreened covariates: child sex, child age, child birth order, mother’s age, mother’s height, mother’s education, household food security, number of children < 18 years old in the household, number of people living in the compound, distance (in minutes) to the primary water source, household materials (floor, roof), asset-based household wealth (electricity, clock, working radio, working black/white or color television, bicycle, motorcycle, sewing machine, mobile phone, land phone, stove, number of cows, number of goats, number of dogs, and number of poultry), treatment arm, prior length-for-age Z-score and weight-for-age Z-score, and month of measurement. See Supplementary Table 1 for more details. | | | | | | | |
| ^2^--^ indicates hazard ratio not calculated due to data sparsity. | | | | | | | |
| ^3^Asterisk denotes level of significance: *** <0.001, ** <0.05, * <0.2 | | | | | | | |

Supplementary Table 8. **EED and Child Development in Kenya**

| Outcome Subdomain | Exposure | n | Q1 | Q3 | Adj. Diff (95% CI)^1^ | Adj. P-value^1,2^ | FDR Corrected P-value^1,2^ |
| --- | --- | --- | --- | --- | --- | --- | --- |
| WHO Motor Milestones (Year 1) | | | | | | | |
| Milestones 2,4,5,6^3^ | alpha-1 antitrypsin (6 mo) | 1001 | -1.30 | -0.04 | 0.04 (-0.04, 0.13) | 0.32 | 0.89 |
| Sum Total | alpha-1 antitrypsin (6 mo) | 1001 | -1.30 | -0.04 | 0.01 (-0.07, 0.10) | 0.80 | 0.89 |
| Milestones 2,4,5,6^3^ | myeloperoxidase (6 mo) | 1002 | 7.41 | 8.90 | 0.02 (-0.05, 0.09) | 0.60 | 0.89 |
| Sum Total | myeloperoxidase (6 mo) | 1002 | 7.41 | 8.90 | 0.01 (-0.07, 0.09) | 0.82 | 0.89 |
| Milestones 2,4,5,6^3^ | neopterin (6 mo) | 1003 | 7.11 | 7.96 | 0.07 (-0.02, 0.16) | 0.16* | 0.88 |
| Sum Total | neopterin (6 mo) | 1003 | 7.11 | 7.96 | 0.07 (-0.03, 0.17) | 0.18* | 0.88 |
| Milestones 2,4,5,6^3^ | lactulose (6 mo) | 1083 | -2.24 | -0.85 | 0.01 (-0.06, 0.08) | 0.71 | 0.89 |
| Sum Total | lactulose (6 mo) | 1083 | -2.24 | -0.85 | 0.01 (-0.07, 0.08) | 0.89 | 0.89 |
| Milestones 2,4,5,6^3^ | mannitol (6 mo) | 1083 | -0.06 | 1.29 | 0.02 (-0.05, 0.09) | 0.57 | 0.89 |
| Sum Total | mannitol (6 mo) | 1083 | -0.06 | 1.29 | 0.02 (-0.06, 0.10) | 0.62 | 0.89 |
| Extended Ages and Stages (Year 2) | | | | | | | |
| Communication | alpha-1 antitrypsin (6 mo) | 796 | -1.27 | -0.01 | 0.10 (0.01, 0.19) | 0.03** | 0.62 |
| Motor | alpha-1 antitrypsin (6 mo) | 816 | -1.27 | -0.02 | -0.01 (-0.10, 0.08) | 0.89 | 0.97 |
| Personal Social | alpha-1 antitrypsin (6 mo) | 818 | -1.27 | -0.02 | 0.04 (-0.06, 0.13) | 0.43 | 0.87 |
| Combined | alpha-1 antitrypsin (6 mo) | 816 | -1.27 | -0.02 | 0.06 (-0.03, 0.15) | 0.22 | 0.87 |
| Communication | myeloperoxidase (6 mo) | 797 | 7.46 | 8.96 | -0.02 (-0.11, 0.06) | 0.60 | 0.87 |
| Motor | myeloperoxidase (6 mo) | 817 | 7.45 | 8.95 | 0.01 (-0.07, 0.10) | 0.76 | 0.90 |
| Personal Social | myeloperoxidase (6 mo) | 819 | 7.43 | 8.94 | -0.03 (-0.12, 0.06) | 0.52 | 0.87 |
| Combined | myeloperoxidase (6 mo) | 817 | 7.45 | 8.95 | -0.02 (-0.11, 0.06) | 0.60 | 0.87 |
| Communication | neopterin (6 mo) | 798 | 7.12 | 7.96 | 0.07 (-0.06, 0.20) | 0.29 | 0.87 |
| Motor | neopterin (6 mo) | 818 | 7.12 | 7.96 | 0.03 (-0.09, 0.15) | 0.67 | 0.87 |
| Personal Social | neopterin (6 mo) | 820 | 7.12 | 7.96 | 0.04 (-0.10, 0.18) | 0.59 | 0.87 |
| Combined | neopterin (6 mo) | 818 | 7.12 | 7.96 | 0.07 (-0.06, 0.20) | 0.29 | 0.87 |
| Communication | lactulose (6 mo) | 861 | -2.25 | -0.85 | 0.10 (-0.07, 0.28) | 0.26 | 0.87 |
| Motor | lactulose (6 mo) | 862 | -2.25 | -0.85 | -0.04 (-0.12, 0.03) | 0.28 | 0.87 |
| Personal Social | lactulose (6 mo) | 865 | -2.25 | -0.85 | 0.08 (-0.01, 0.16) | 0.08* | 0.87 |
| Combined | lactulose (6 mo) | 861 | -2.25 | -0.85 | 0.04 (-0.11, 0.19) | 0.59 | 0.87 |
| Communication | mannitol (6 mo) | 861 | -0.05 | 1.31 | 0.06 (-0.02, 0.14) | 0.15* | 0.87 |
| Motor | mannitol (6 mo) | 862 | -0.05 | 1.31 | 0.01 (-0.07, 0.08) | 0.90 | 0.97 |
| Personal Social | mannitol (6 mo) | 865 | -0.05 | 1.31 | 0.15 (0.04, 0.25) | 0.01*** | 0.26 |
| Combined | mannitol (6 mo) | 861 | -0.05 | 1.31 | 0.05 (-0.03, 0.13) | 0.20* | 0.87 |
| Communication | alpha-1 antitrypsin (17 mo) | 791 | -1.78 | -0.68 | 0.02 (-0.07, 0.11) | 0.64 | 0.87 |
| Motor | alpha-1 antitrypsin (17 mo) | 777 | -1.80 | -0.68 | 0.00 (-0.07, 0.07) | 0.97 | 0.98 |
| Personal Social | alpha-1 antitrypsin (17 mo) | 1010 | -1.79 | -0.68 | 0.06 (-0.05, 0.17) | 0.31 | 0.87 |
| Combined | alpha-1 antitrypsin (17 mo) | 760 | -1.80 | -0.68 | 0.04 (-0.07, 0.15) | 0.45 | 0.87 |
| Communication | myeloperoxidase (17 mo) | 791 | 6.52 | 7.90 | 0.04 (-0.11, 0.18) | 0.64 | 0.87 |
| Motor | myeloperoxidase (17 mo) | 777 | 6.52 | 7.90 | 0.00 (-0.08, 0.08) | 0.96 | 0.98 |
| Personal Social | myeloperoxidase (17 mo) | 1008 | 6.53 | 7.87 | 0.06 (-0.01, 0.14) | 0.11* | 0.87 |
| Combined | myeloperoxidase (17 mo) | 760 | 6.54 | 7.90 | 0.06 (-0.03, 0.14) | 0.18* | 0.87 |
| Communication | neopterin (17 mo) | 791 | 5.25 | 6.95 | 0.02 (-0.07, 0.12) | 0.62 | 0.87 |
| Motor | neopterin (17 mo) | 777 | 5.25 | 6.94 | 0.04 (-0.07, 0.15) | 0.48 | 0.87 |
| Personal Social | neopterin (17 mo) | 1011 | 5.26 | 6.95 | 0.02 (-0.07, 0.11) | 0.63 | 0.87 |
| Combined | neopterin (17 mo) | 760 | 5.25 | 6.94 | 0.02 (-0.08, 0.12) | 0.71 | 0.88 |
| Communication | lactulose (17 mo) | 831 | -2.68 | -0.98 | 0.00 (-0.15, 0.16) | 0.98 | 0.98 |
| Motor | lactulose (17 mo) | 831 | -2.68 | -0.98 | -0.01 (-0.08, 0.05) | 0.67 | 0.87 |
| Personal Social | lactulose (17 mo) | 1078 | -2.77 | -0.93 | 0.02 (-0.07, 0.11) | 0.72 | 0.88 |
| Combined | lactulose (17 mo) | 831 | -2.68 | -0.98 | 0.04 (-0.08, 0.15) | 0.52 | 0.87 |
| Communication | mannitol (17 mo) | 831 | -0.42 | 1.18 | 0.05 (-0.05, 0.14) | 0.34 | 0.87 |
| Motor | mannitol (17 mo) | 831 | -0.42 | 1.18 | 0.01 (-0.07, 0.08) | 0.87 | 0.97 |
| Personal Social | mannitol (17 mo) | 1078 | -0.42 | 1.24 | -0.02 (-0.09, 0.05) | 0.61 | 0.87 |
| Combined | mannitol (17 mo) | 831 | -0.42 | 1.18 | 0.03 (-0.08, 0.13) | 0.63 | 0.87 |
|  | | | | | | | |
|  | | | | | | | |

Abbreviations: EED, environmental enteric dysfunction; mo, months; Q1, first quartile; Q3, third quartile; Adj Diff, adjusted difference in outcome between the 25^th^ and 75^th^ percentile of exposure; Adj P-value, P-value of adjusted difference between the 25^th^ and 75^th^ percentile of exposure; FDR, false discovery rate

|  |  |
| --- | --- |
| ^1^Adjusted for prespecified and prescreened covariates: child sex, child age, child birth order, mother’s age, mother’s height, mother’s education, household food security, number of children < 18 years old in the household, number of people living in the compound, distance (in minutes) to the primary water source, household materials (floor, roof), asset-based household wealth (electricity, clock, working radio, working black/white or color television, bicycle, motorcycle, sewing machine, mobile phone, land phone, stove, number of cows, number of goats, number of dogs, and number of poultry), treatment arm, prior length-for-age Z-score and weight-for-age Z-score, month of measurement, mother’s perceived stress (only for year 2 outcomes), and mother’s depressive symptoms (only for year 2 outcomes). See Supplementary Table 1 for more details. | |
| ^2^Asterisk denotes level of significance: *** <0.001, ** <0.05, * <0.2 | |
| ^3^Milestones 2,4,5,6 correspond to hands-and-knees crawling, walking with assistance, standing alone, and walking alone, respectively. | |

| Supplementary Table 9. **Exclusive Lactation Sensitivity Analysis in Kenya** | | | | | | | |
| --- | --- | --- | --- | --- | --- | --- | --- |
|  |  | Exclusive Lactation Variable Included | | | Exclusive Lactation Variable Not Included | | |
| Outcome Subdomain | Exposure | n^1^ | Adj. Diff (95% CI)^1^ | Adj. P-value^1,2^ | n^1^ | Adj. Diff (95% CI)^1^ | Adj. P-value^1,2^ |
| WHO Motor Milestones (Year 1) | | | | | | | |
| Milestones 2,4,5,6^3^ | alpha-1 antitrypsin (6 mo) | 1000 | 0.04 (-0.04, 0.13) | 0.31 | 1001 | 0.04 (-0.04, 0.13) | 0.32 |
| Sum Total | alpha-1 antitrypsin (6 mo) | 1000 | 0.01 (-0.07, 0.10) | 0.80 | 1001 | 0.01 (-0.07, 0.10) | 0.80 |
| Milestones 2,4,5,6^3^ | myeloperoxidase (6 mo) | 1001 | 0.02 (-0.05, 0.09) | 0.61 | 1002 | 0.02 (-0.05, 0.09) | 0.60 |
| Sum Total | myeloperoxidase (6 mo) | 1001 | 0.01 (-0.07, 0.09) | 0.84 | 1002 | 0.01 (-0.07, 0.09) | 0.82 |
| Milestones 2,4,5,6^3^ | neopterin (6 mo) | 1002 | 0.06 (-0.03, 0.16) | 0.17* | 1003 | 0.07 (-0.02, 0.16) | 0.16* |
| Sum Total | neopterin (6 mo) | 1002 | 0.07 (-0.03, 0.17) | 0.19* | 1003 | 0.07 (-0.03, 0.17) | 0.18* |
| Milestones 2,4,5,6^3^ | lactulose (6 mo) | 1082 | 0.02 (-0.05, 0.09) | 0.64 | 1083 | 0.01 (-0.06, 0.08) | 0.71 |
| Sum Total | lactulose (6 mo) | 1082 | 0.01 (-0.07, 0.08) | 0.86 | 1083 | 0.01 (-0.07, 0.08) | 0.89 |
| Milestones 2,4,5,6^3^ | mannitol (6 mo) | 1082 | 0.02 (-0.05, 0.09) | 0.51 | 1083 | 0.02 (-0.05, 0.09) | 0.57 |
| Sum Total | mannitol (6 mo) | 1082 | 0.02 (-0.05, 0.10) | 0.59 | 1083 | 0.02 (-0.06, 0.10) | 0.62 |
| Extended Ages and Stages (Year 2) | | | | | | | |
| Communication | alpha-1 antitrypsin (6 mo) | 796 | 0.10 (0.01, 0.19) | 0.03** | 796 | 0.10 (0.01, 0.19) | 0.03** |
| Motor | alpha-1 antitrypsin (6 mo) | 820 | -0.01 (-0.10, 0.08) | 0.82 | 816 | -0.01 (-0.10, 0.08) | 0.89 |
| Personal Social | alpha-1 antitrypsin (6 mo) | 822 | 0.04 (-0.05, 0.14) | 0.36 | 818 | 0.04 (-0.06, 0.13) | 0.43 |
| Combined | alpha-1 antitrypsin (6 mo) | 820 | 0.05 (-0.05, 0.14) | 0.33 | 816 | 0.06 (-0.03, 0.15) | 0.22 |
| Communication | myeloperoxidase (6 mo) | 797 | -0.03 (-0.12, 0.06) | 0.48 | 797 | -0.02 (-0.11, 0.06) | 0.60 |
| Motor | myeloperoxidase (6 mo) | 821 | 0.01 (-0.08, 0.09) | 0.90 | 817 | 0.01 (-0.07, 0.10) | 0.76 |
| Personal Social | myeloperoxidase (6 mo) | 823 | -0.04 (-0.13, 0.05) | 0.41 | 819 | -0.03 (-0.12, 0.06) | 0.52 |
| Combined | myeloperoxidase (6 mo) | 821 | -0.03 (-0.12, 0.06) | 0.48 | 817 | -0.02 (-0.11, 0.06) | 0.60 |
| Communication | neopterin (6 mo) | 798 | 0.11 (-0.03, 0.25) | 0.13* | 798 | 0.07 (-0.06, 0.20) | 0.29 |
| Motor | neopterin (6 mo) | 822 | 0.04 (-0.08, 0.16) | 0.54 | 818 | 0.03 (-0.09, 0.15) | 0.67 |
| Personal Social | neopterin (6 mo) | 824 | 0.05 (-0.11, 0.20) | 0.58 | 820 | 0.04 (-0.10, 0.18) | 0.59 |
| Combined | neopterin (6 mo) | 822 | 0.08 (-0.06, 0.22) | 0.28 | 818 | 0.07 (-0.06, 0.20) | 0.29 |
| Communication | lactulose (6 mo) | 860 | 0.09 (-0.08, 0.27) | 0.29 | 861 | 0.10 (-0.07, 0.28) | 0.26 |
| Motor | lactulose (6 mo) | 861 | -0.02 (-0.10, 0.05) | 0.55 | 862 | -0.04 (-0.12, 0.03) | 0.28 |
| Personal Social | lactulose (6 mo) | 864 | 0.09 (0.00, 0.17) | 0.05* | 865 | 0.08 (-0.01, 0.16) | 0.08* |
| Combined | lactulose (6 mo) | 860 | 0.06 (-0.09, 0.21) | 0.46 | 861 | 0.04 (-0.11, 0.19) | 0.59 |
| Communication | mannitol (6 mo) | 860 | 0.06 (-0.02, 0.14) | 0.12* | 861 | 0.06 (-0.02, 0.14) | 0.15* |
| Motor | mannitol (6 mo) | 861 | 0.06 (-0.10, 0.21) | 0.50 | 862 | 0.01 (-0.07, 0.08) | 0.90 |
| Personal Social | mannitol (6 mo) | 864 | 0.15 (0.05, 0.26) | <0.01*** | 865 | 0.15 (0.04, 0.25) | 0.01** |
| Combined | mannitol (6 mo) | 860 | 0.07 (-0.02, 0.15) | 0.11* | 861 | 0.05 (-0.03, 0.13) | 0.20 |

| Abbreviations: EED, environmental enteric dysfunction; mo, months; Adj Diff, adjusted difference in outcome between the 25^th^ and 75^th^ percentile of exposure; Adj P-value, P-value of adjusted difference between the 25^th^ and 75^th^ percentile of exposure  ^1^Adjusted for prespecified and prescreened covariates: child sex, child age, child birth order, mother’s age, mother’s height, mother’s education, household food security, number of children < 18 years old in the household, number of people living in the compound, distance (in minutes) to the primary water source, household materials (floor, roof), asset-based household wealth (electricity, clock, working radio, working black/white or color television, bicycle, motorcycle, sewing machine, mobile phone, land phone, stove, number of cows, number of goats, number of dogs, and number of poultry), treatment arm, prior length-for-age Z-score and weight-for-age Z-score, month of measurement, mother’s perceived stress (only for year 2 outcomes), and mother’s depressive symptoms (only for year 2 outcomes). See Supplementary Table 1 for more details. |
| --- |
| ^2^Asterisk denotes level of significance: *** <0.01, ** <0.05, * <0.2 |
| ^3^Milestones 2,4,5,6 correspond to hands-and-knees crawling, walking with assistance, standing alone, and walking alone, respectively. |

# Supplementary Appendix A: Spline plots

Each panel of the plots below show the generalized additive model fit between the exposure and outcome denoted in the panel header, with simultaneous confidence intervals for the fitted line. The dashed vertical lines indicate the first and third quartiles of the exposure distribution. The plots are faceted by exposure and outcome subdomain, with the x-axis limits set to the 5th and 95th percentiles of the exposure distribution within each facet to remove tail effects in the outlier ranges of the exposure distributions.


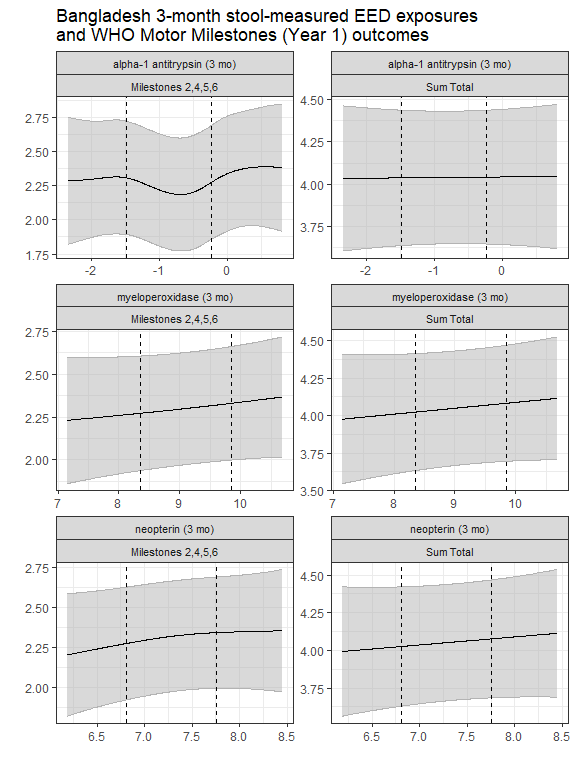

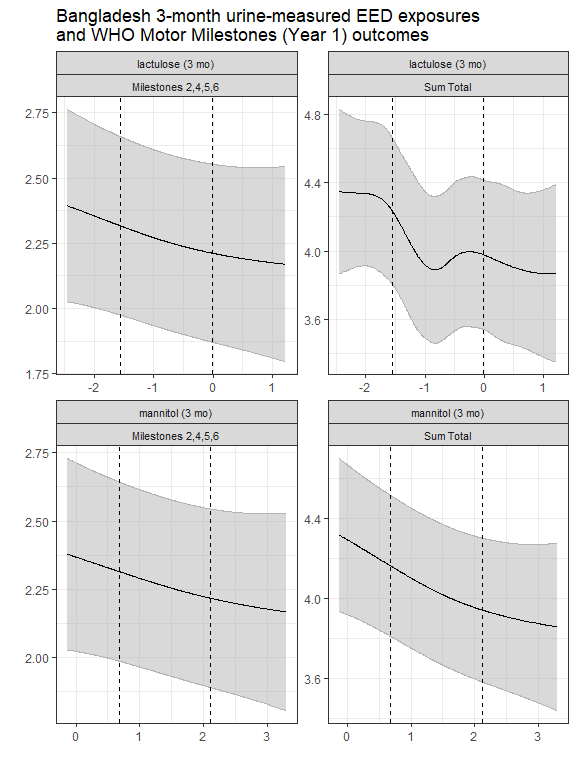

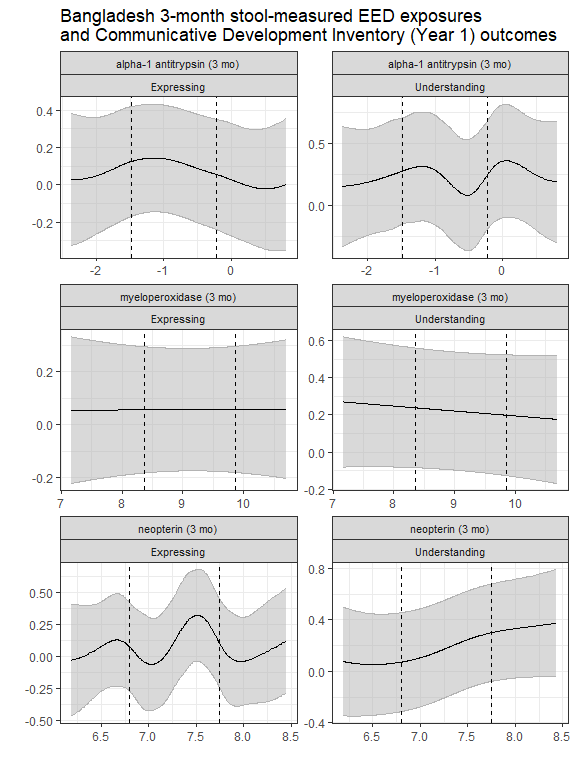

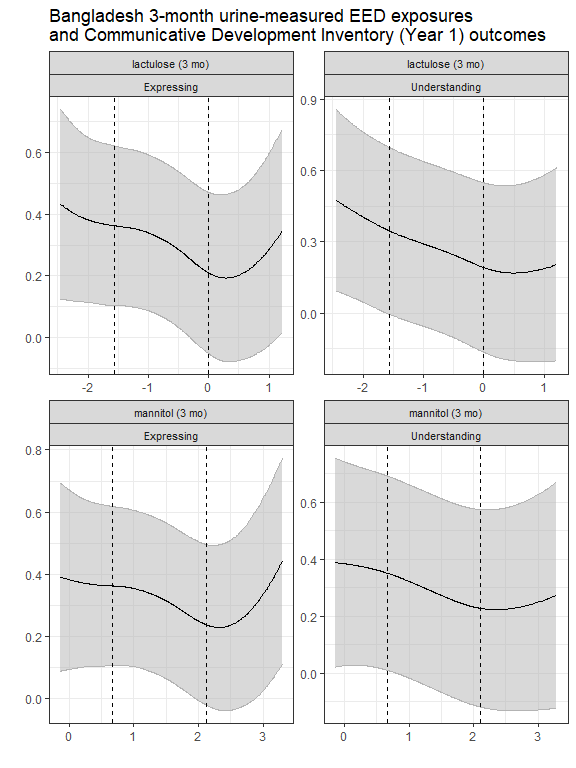


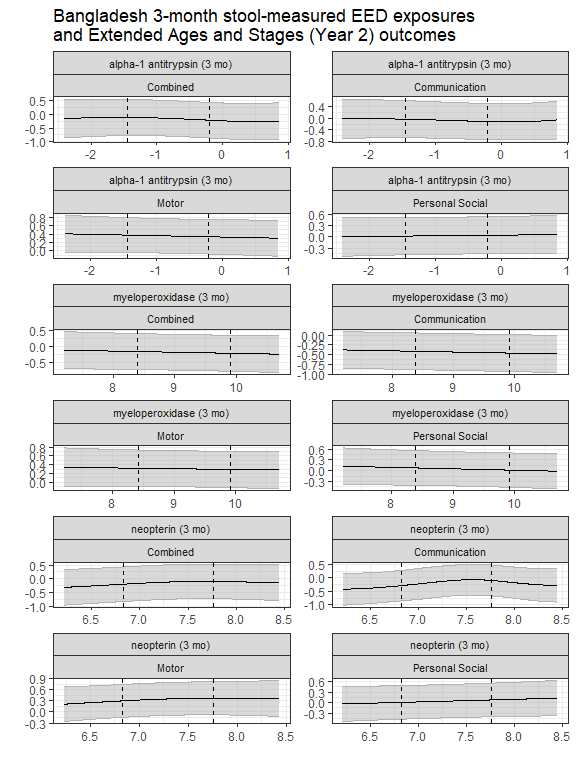

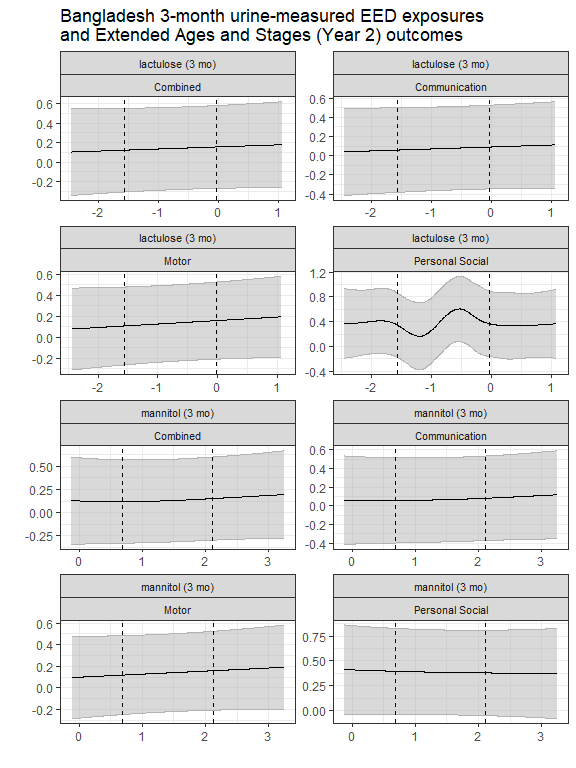

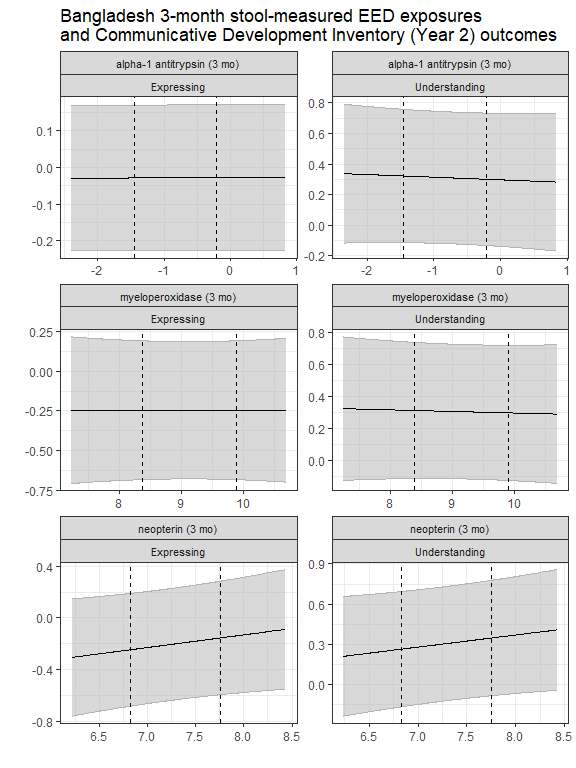

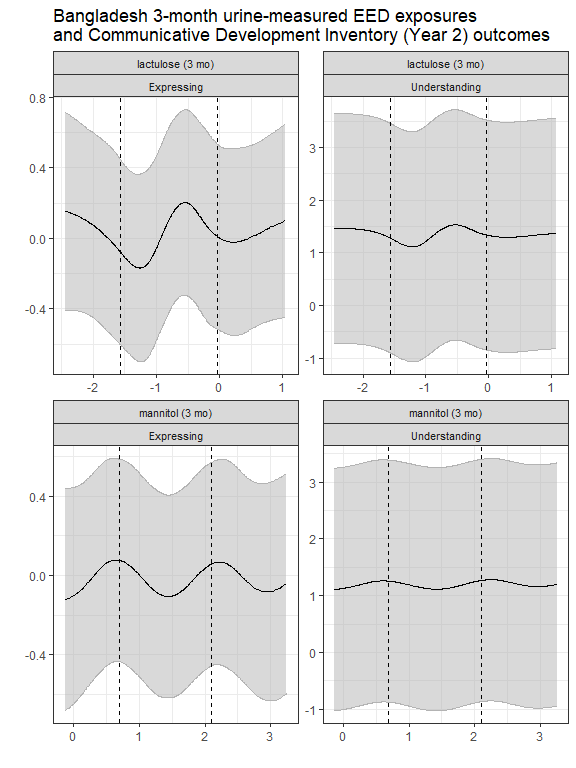

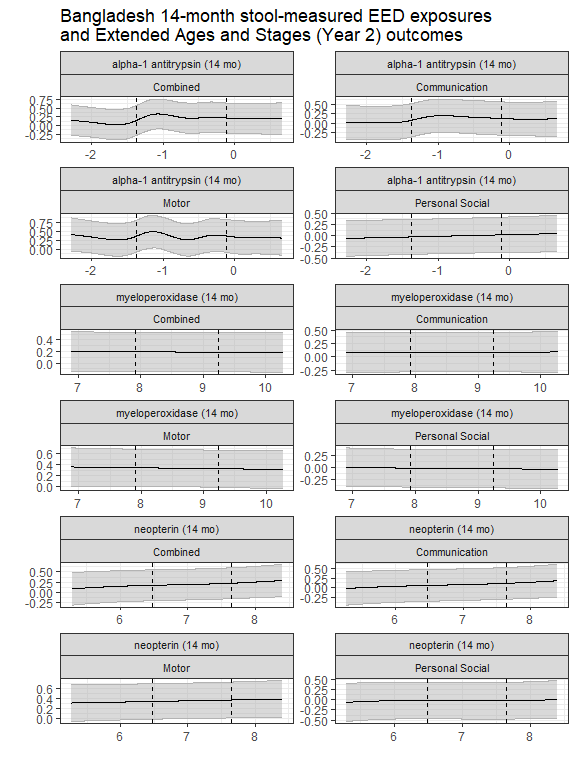

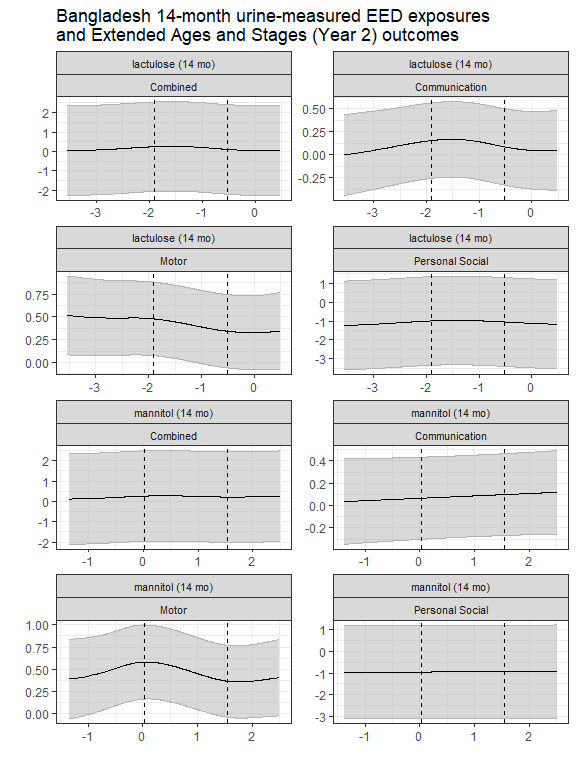

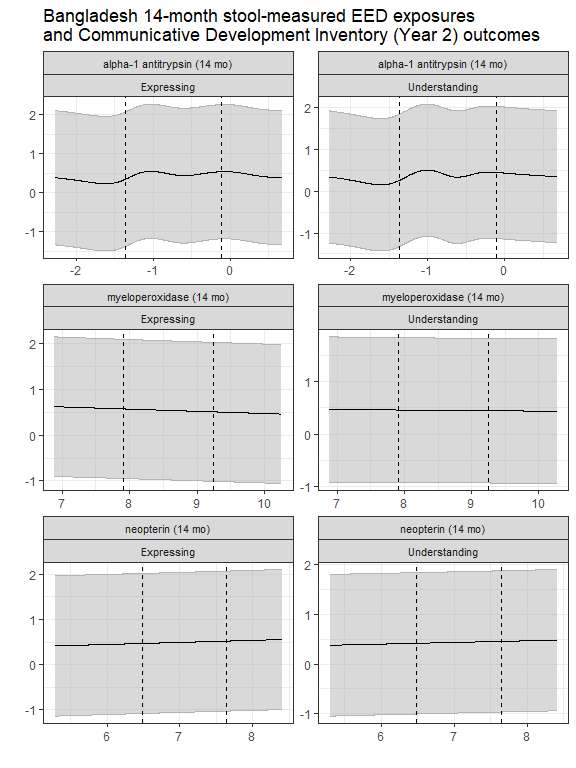

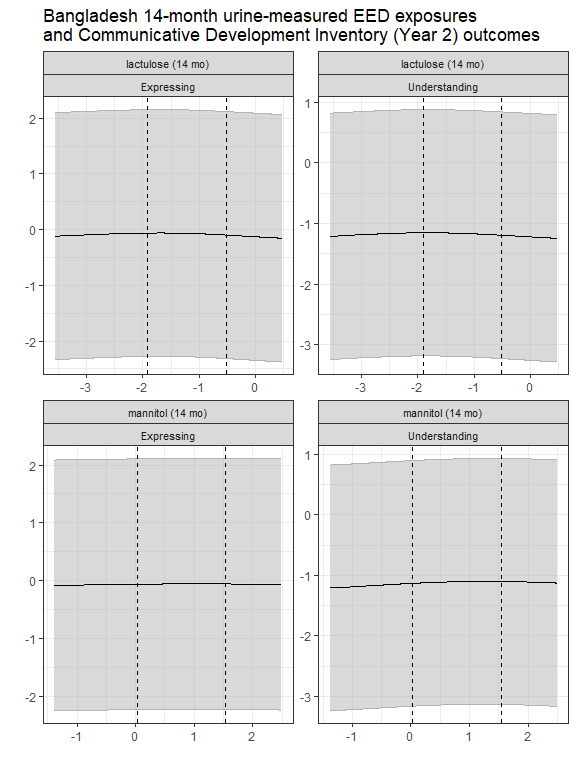


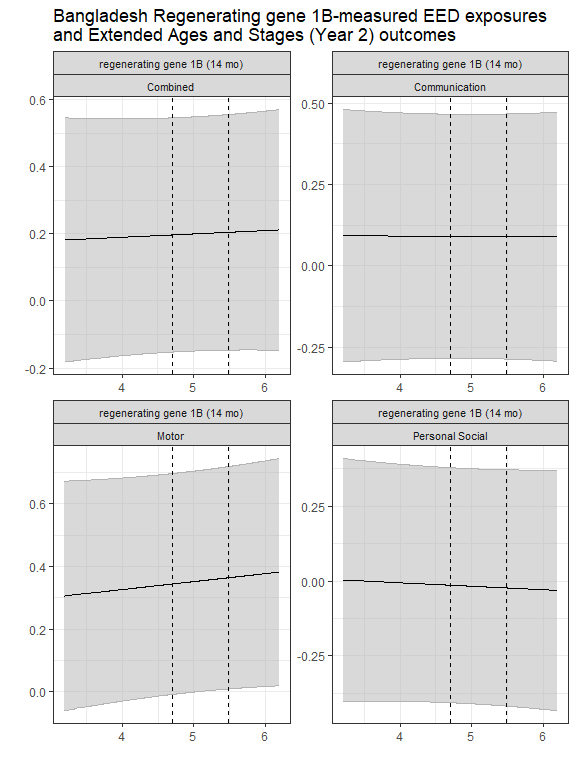

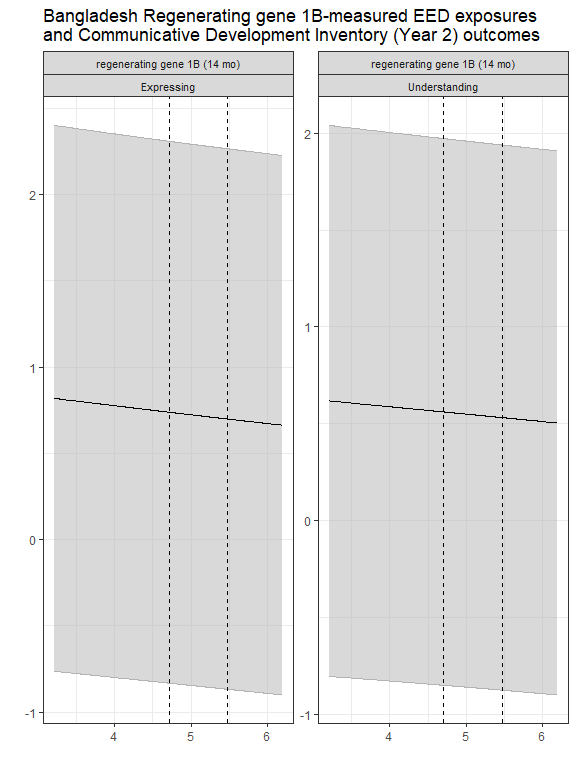


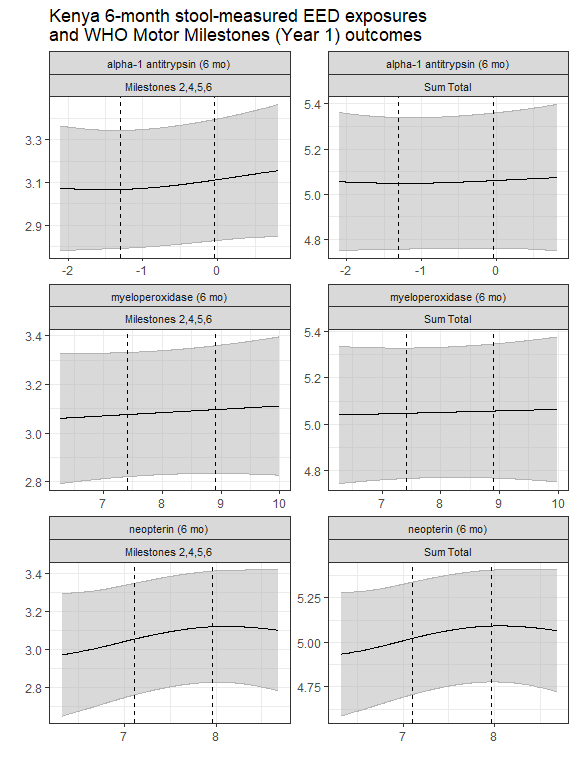

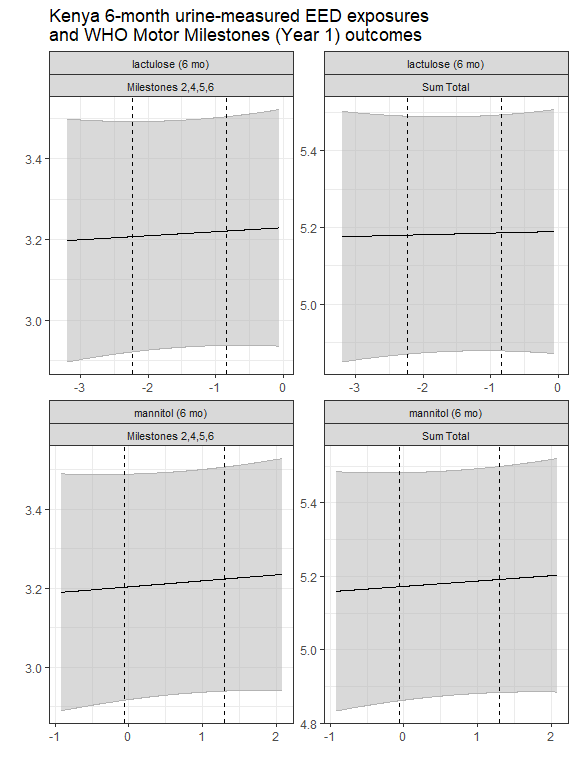


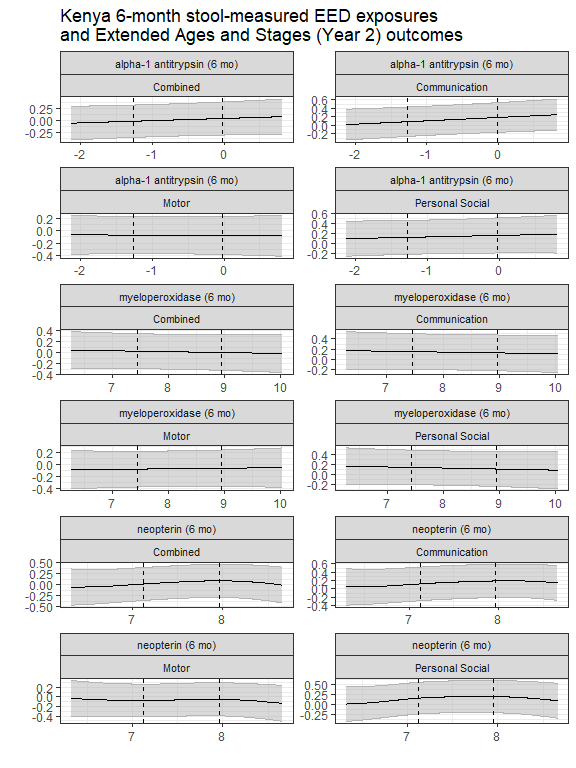

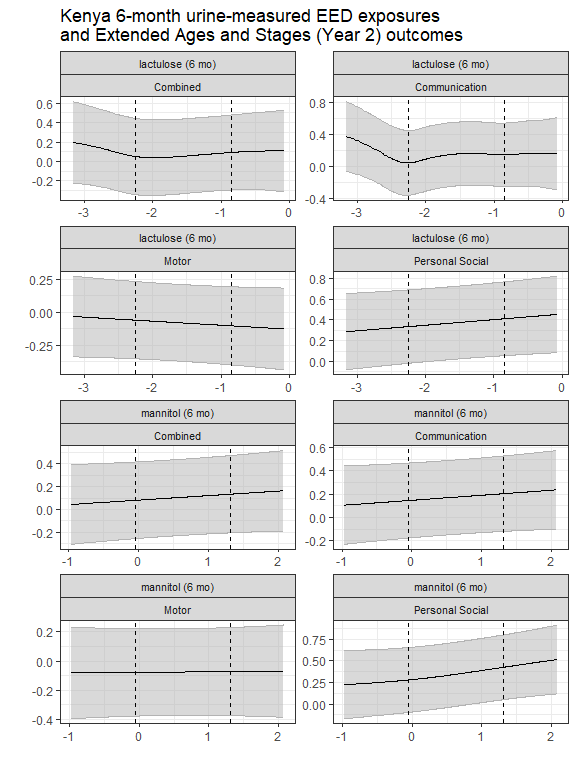

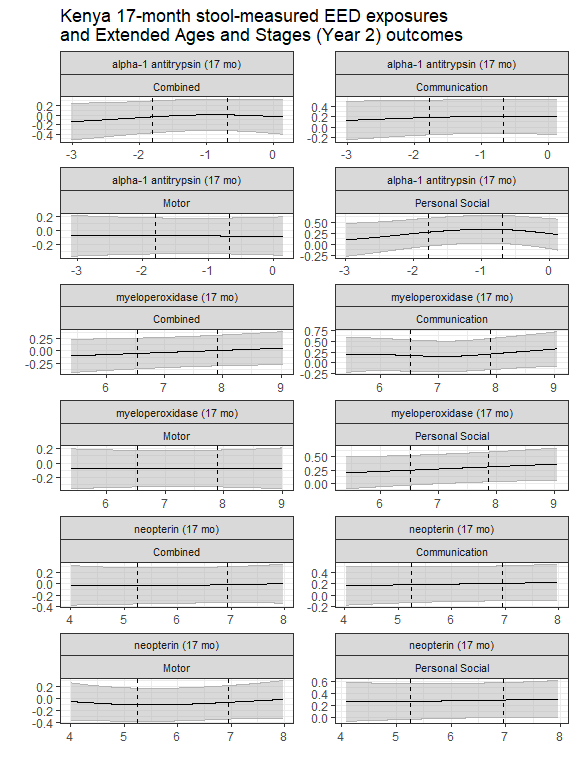

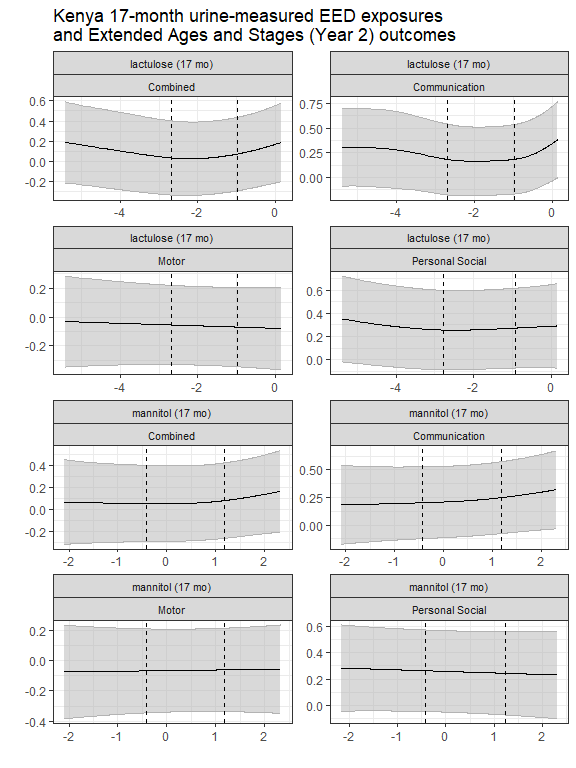


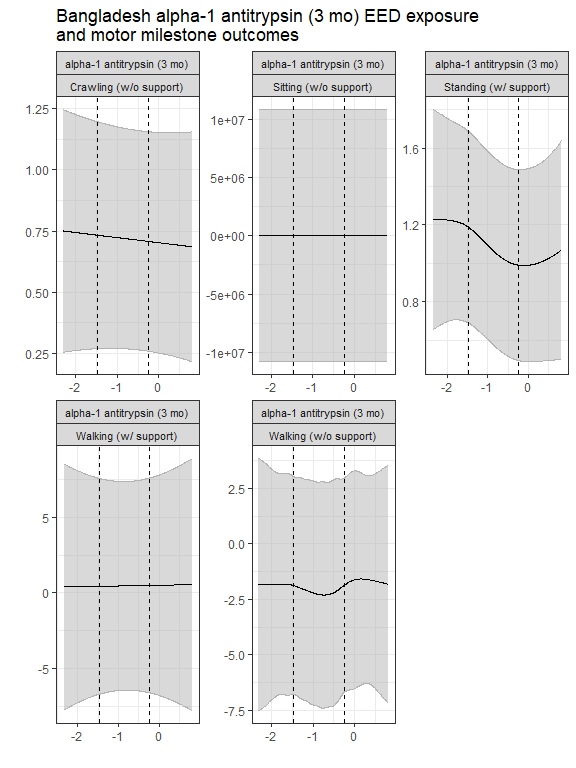

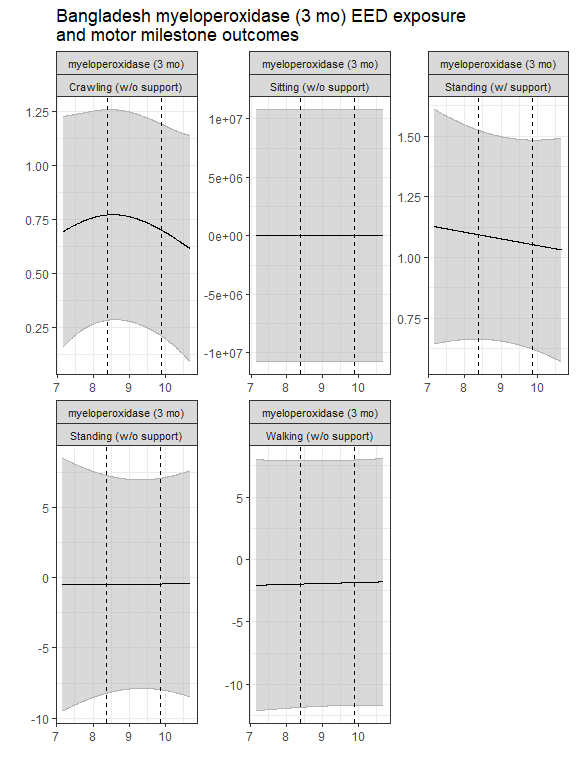

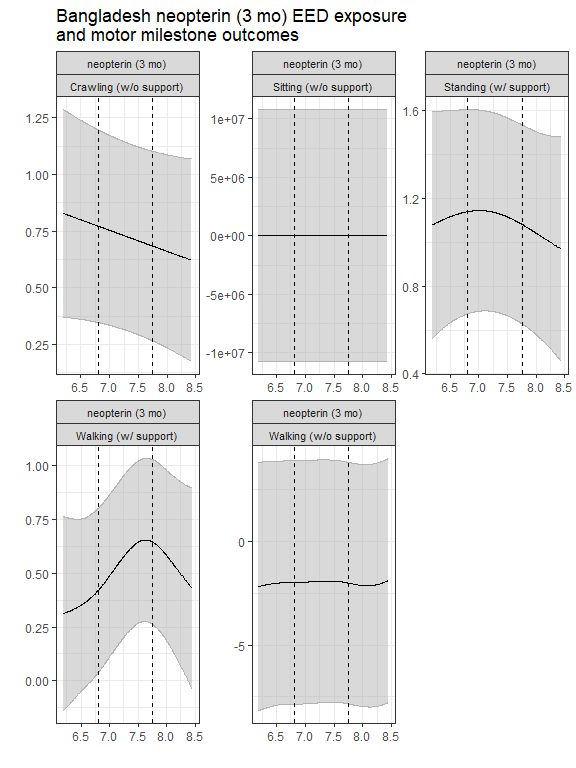

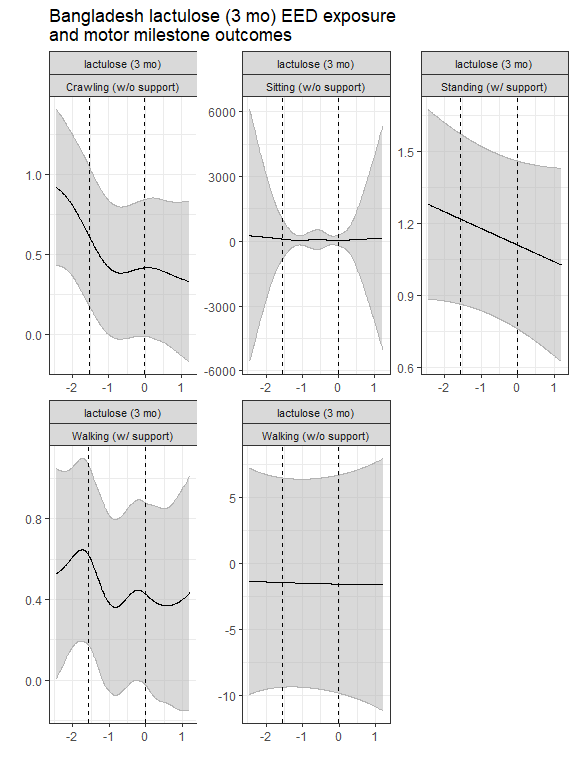

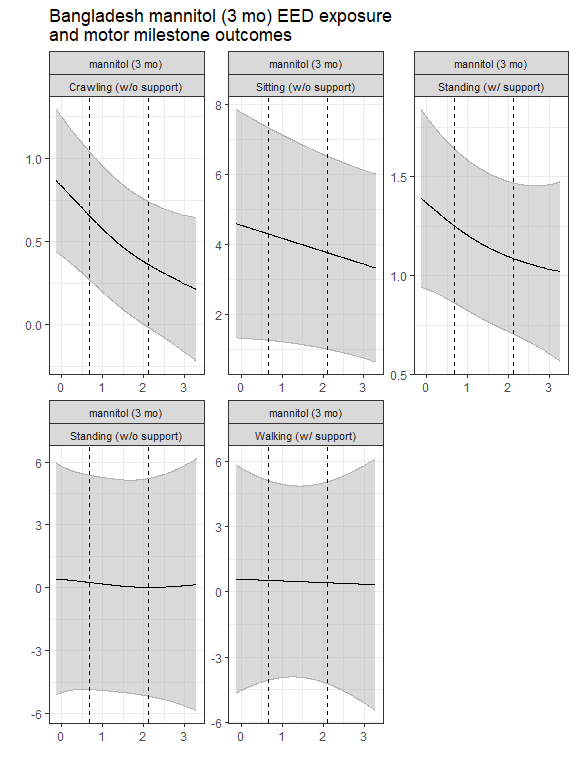

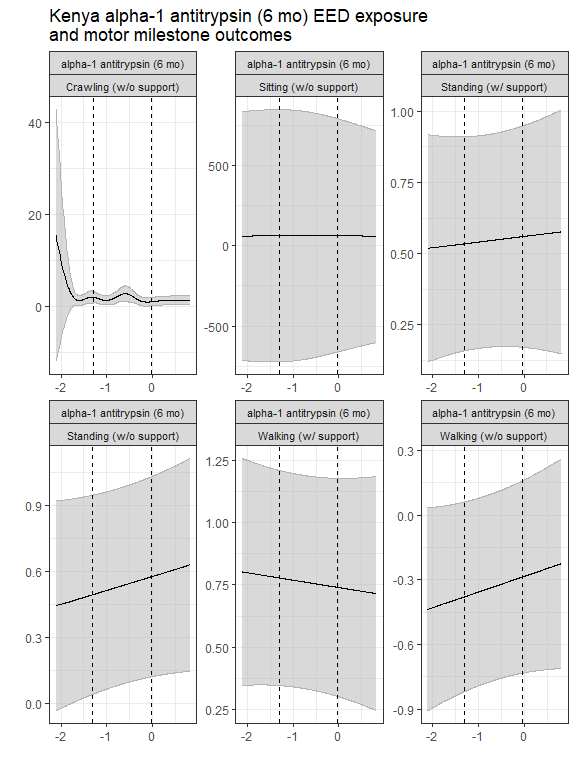

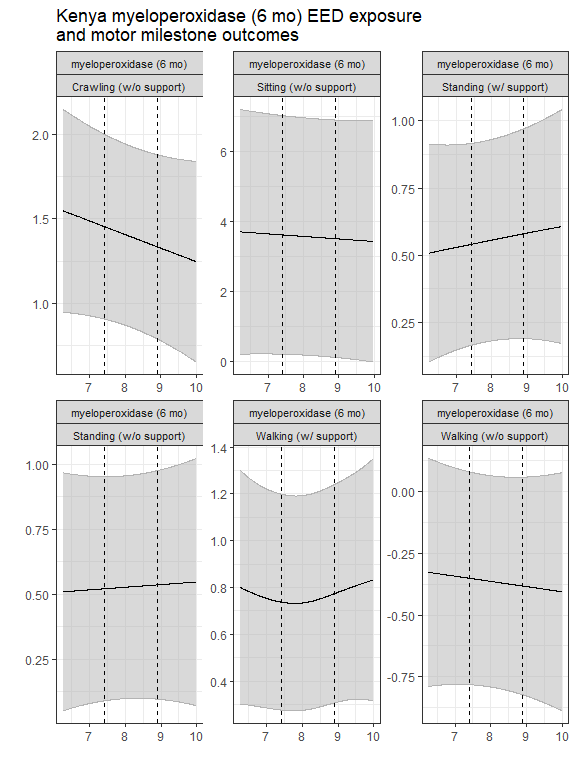

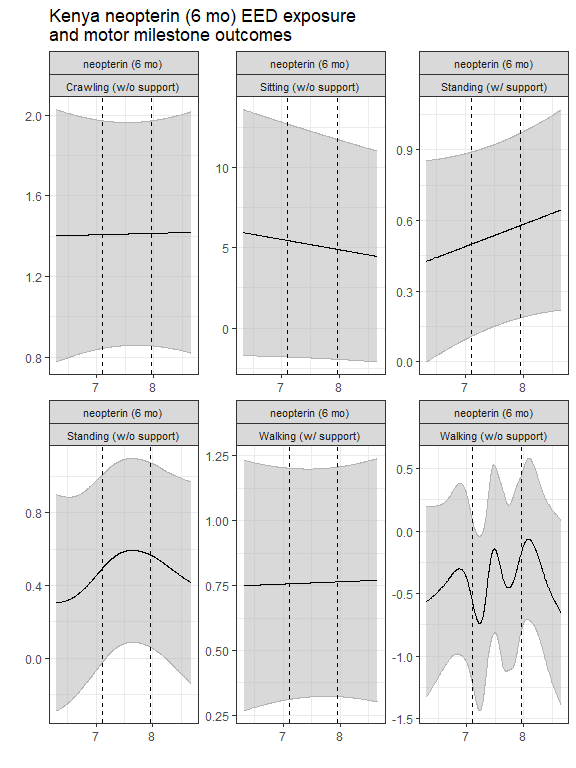

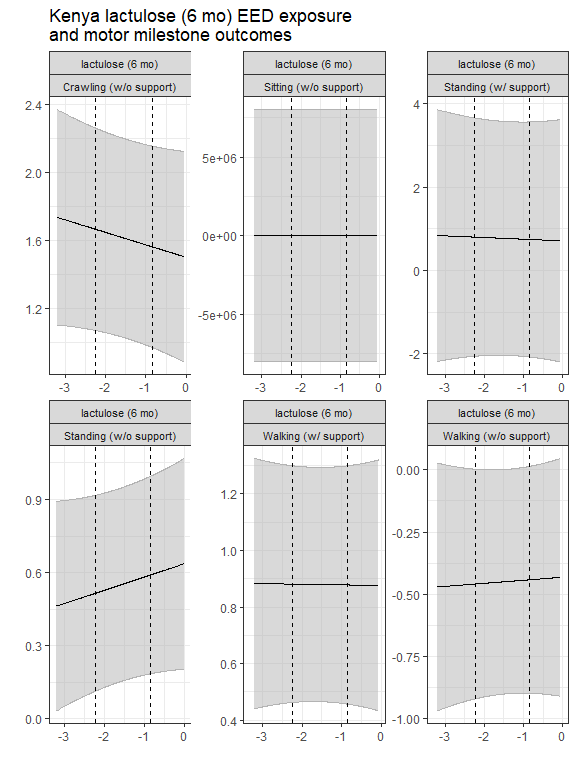

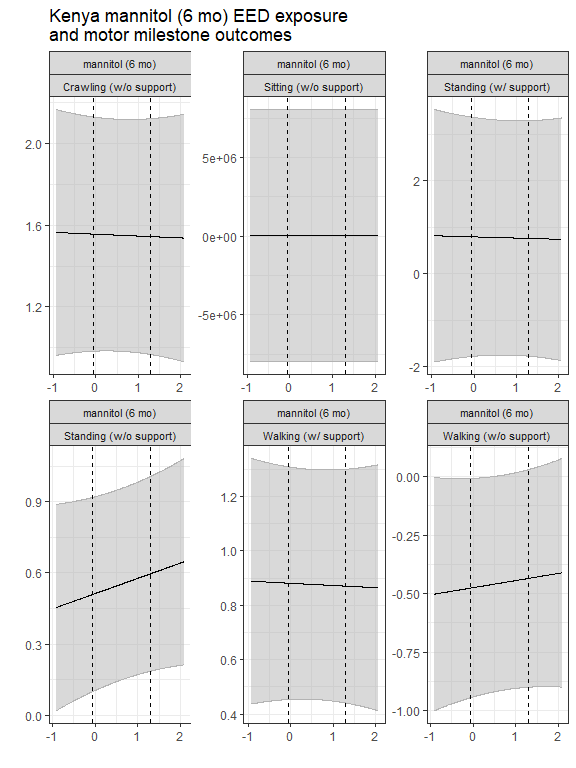


# Supplementary Appendix B: GLM-GAM comparison sensitivity analysis

The plots below show the comparison of the GLM and GAM models for the EED exposures and outcomes. The GLM model is shown in blue, while the GAM model is shown in red. The X-axes are labeled with the exposures and each panel is a different outcome, with the panel title indicating the exposure and outcome. The y-axes are labeled with the model estimates, with the GLM estimates being the coefficient of the exposure and corresponding bootstrap-based 95% confidence interval, while the GAM estimates are the contrast between the 25th and 7th percentiles of the exposure distribution and corresponding bootstrap-based 95% confidence interval. The dashed vertical lines indicate the first and third quartiles of the exposure distribution, with the corresponding confidence interval around the estimated difference.

### Bangladesh cohort
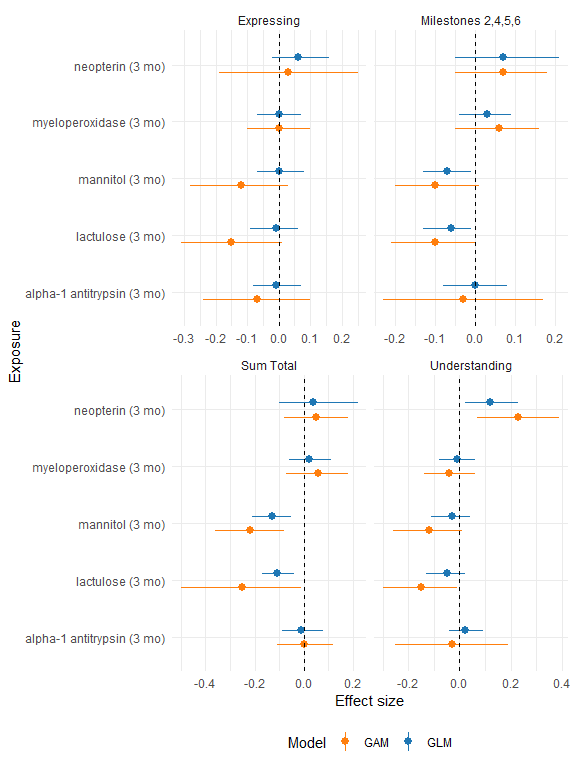

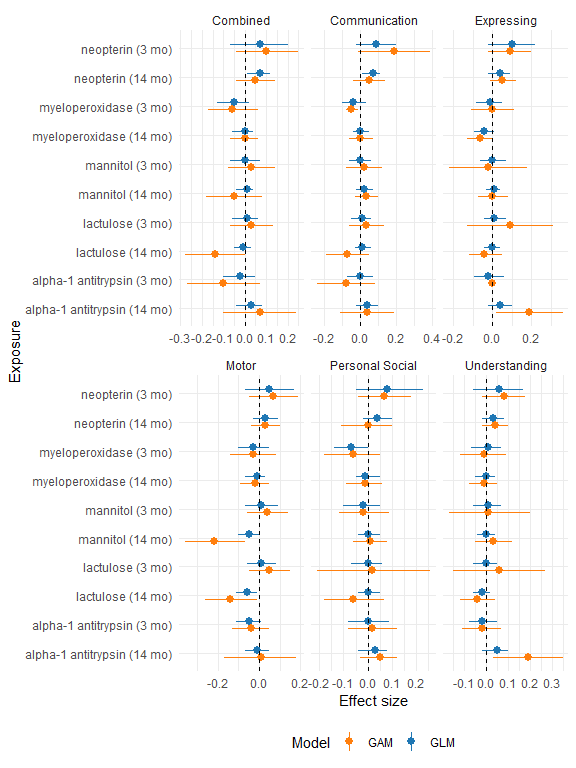

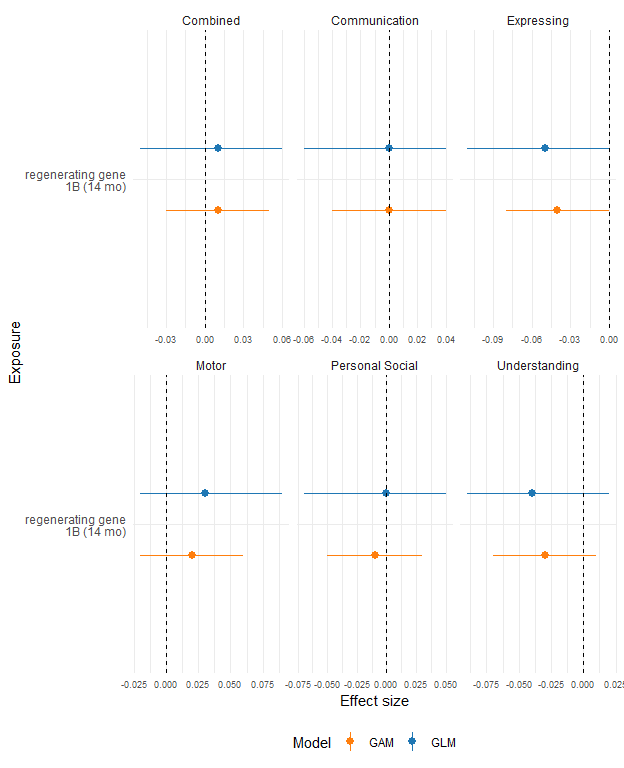


### Kenya cohort
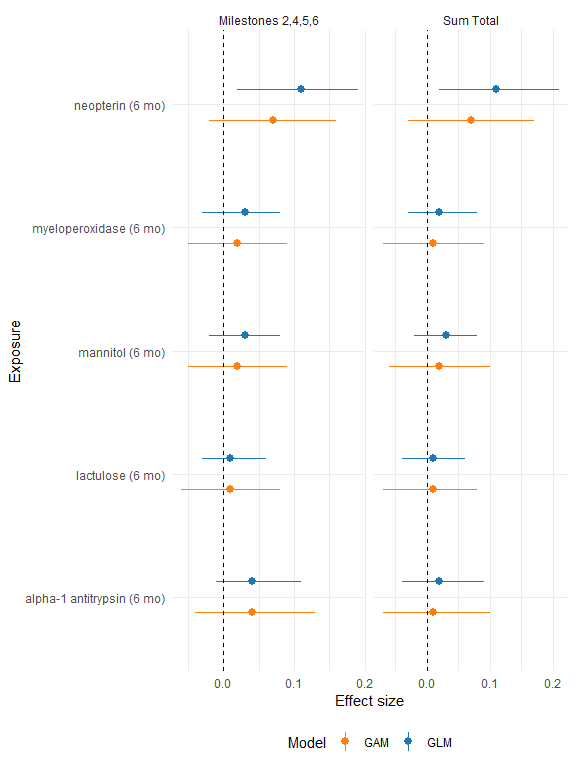

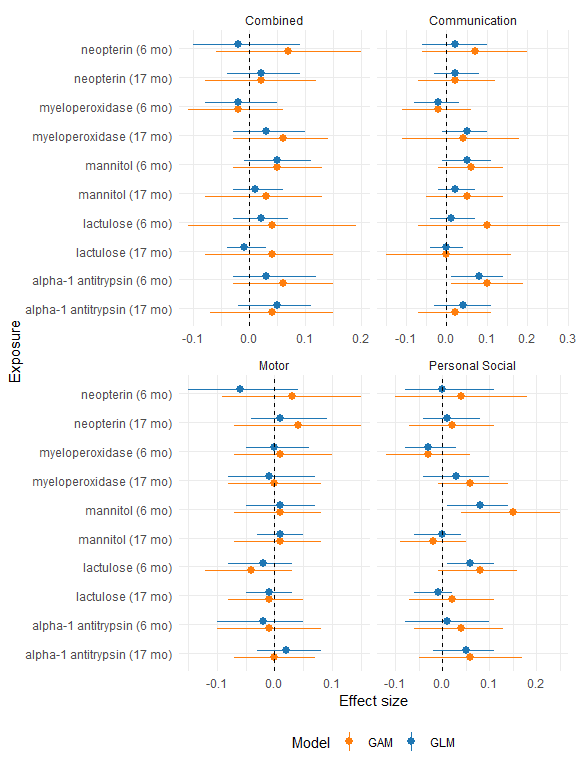


# Supplementary Appendix C: Diarrhea sensitivity analysis

The plots below show the comparison of models fit without prior diarrheal disease included in the model as a confounder (main analysis) versus a sensitivity analysis of models adjusting for prior diarrheal disease.

### Bangladesh cohort
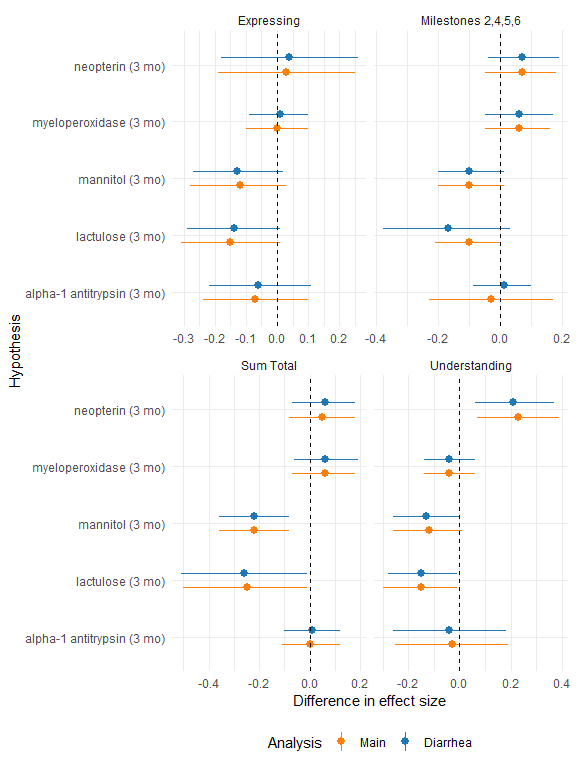

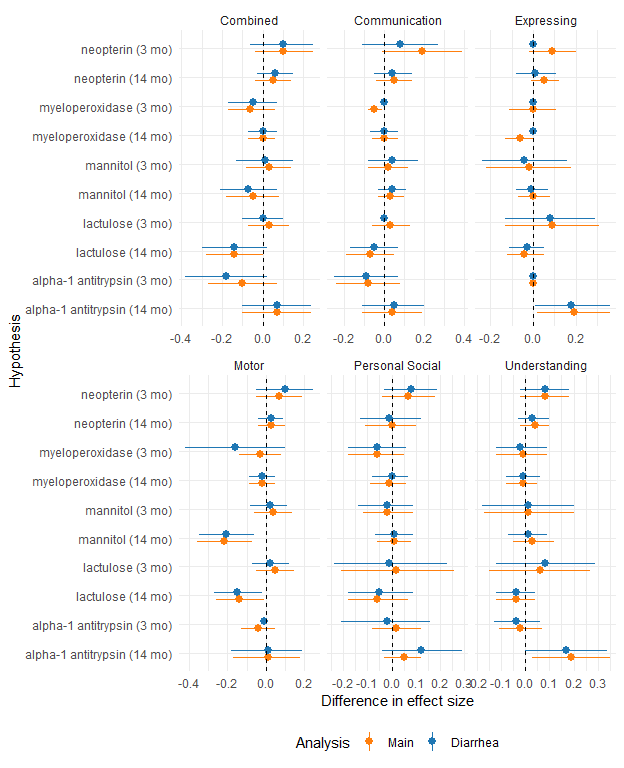


###
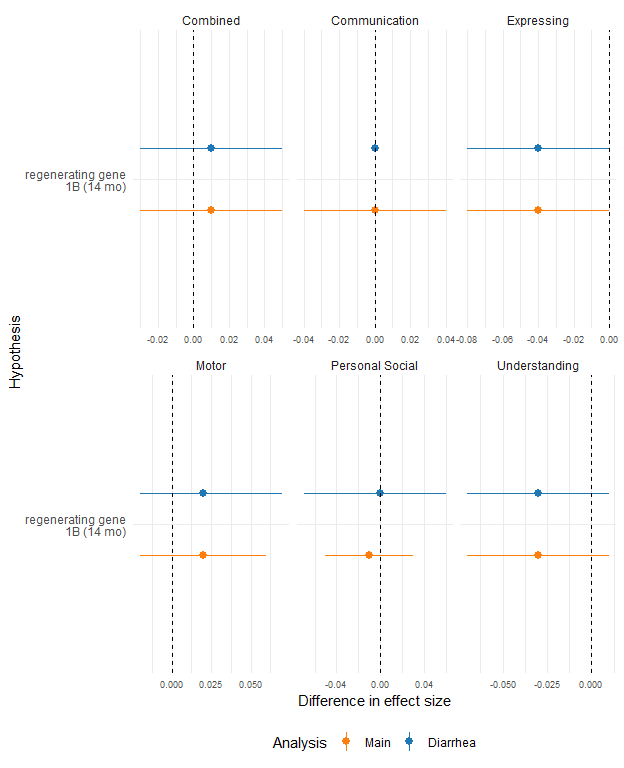


### Kenya cohort
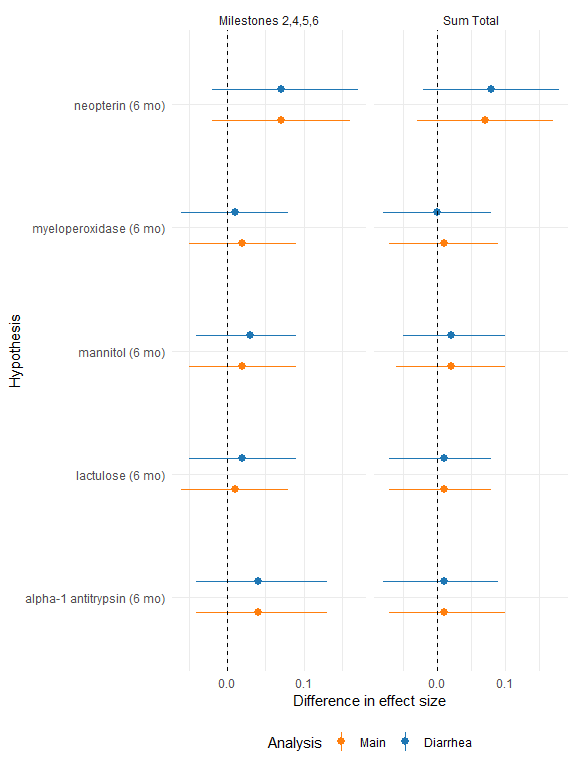

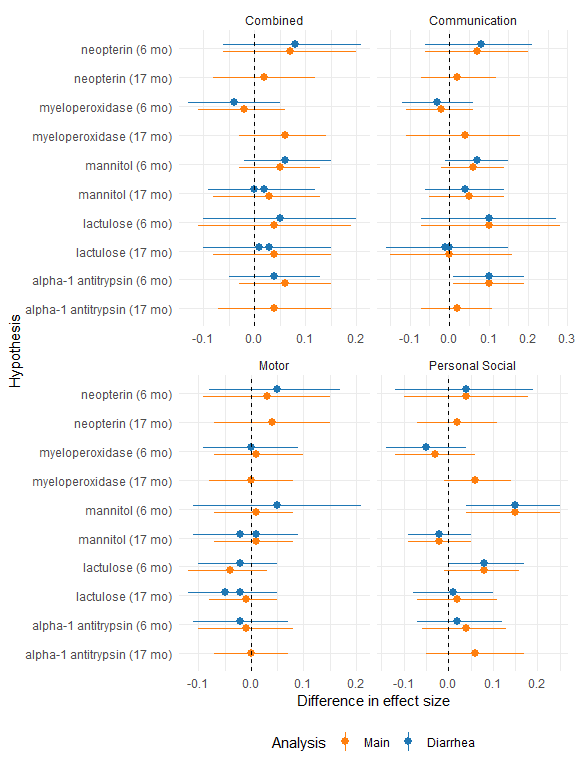

Supplement: multimedia component 1 [file mmc1.docx]
